# OCIAD2 Promotes Cancer Progression via Metabolic Reprogramming in Lung Adenocarcinoma

**DOI:** 10.1021/acs.jproteome.5c00273

**Published:** 2025-07-21

**Authors:** Yi-Hui Huang, Wen-Hsin Chang, Chi-Ya Shen, Kang-Yi Su, Gee-Chen Chang, Jin-Shing Chen, Wen-Yao Lee, Yu-Ju Chen, Sung-Liang Yu

**Affiliations:** † Department of Clinical Laboratory Sciences and Medical Biotechnology, College of Medicine, 33561 National Taiwan University, Taipei 10048, Taiwan; ‡ Graduate Institute of Medical Genomics and Proteomics, College of Medicine, 33561 National Taiwan University, Taipei 100233, Taiwan; § Graduate Institute of Medical Science, College of Medicine, Taipei Medical University, Taipei 11031, Taiwan; ∥ Department of Internal Medicine, 63276 Chung Shan Medical University Hospital, Taichung 40201, Taiwan; ⊥ Department of Surgery, 38005 National Taiwan University Hospital and National Taiwan University College of Medicine, Taipei 10002, Taiwan; # Institute of Chemistry, Academia Sinica, Taipei 11529, Taiwan; 7 Department of Laboratory Medicine, 38006 National Taiwan University Hospital, Taipei 10002, Taiwan; 8 Institute of Medical Device and Imaging, College of Medicine, 33561 National Taiwan University, Taipei 10051, Taiwan; 9 Graduate Institute of Pathology, College of Medicine, 33561 National Taiwan University, Taipei 10051, Taiwan; 10 Graduate School of Advanced Technology, National Taiwan University, Taipei, Taiwan, Taipei 10617, Taiwan

**Keywords:** Ovarian Cancer Immunoreactive Antigen Domain Containing 2, Lung adenocarcinoma, Chromosome-centric Human Proteome Project, Uncharacterized protein existence level 1

## Abstract

Given the limited proteomic insights and high incidence of lung adenocarcinoma, further investigation of uncharacterized proteins in cancer progression remains crucial. In this study, a poorly characterized protein, OCIA domain-containing 2 (OCIAD2), encoded by chromosome 4 was identified as being upregulated in lung adenocarcinoma from our previous proteogenomics data using the Taiwan Cancer Moonshot cohort. OCIAD2 was highly expressed in tumor tissues in 95.5% of lung adenocarcinoma patients in our cohort, with elevated expression correlating with worse survival. Functional studies revealed that the silencing of the OCIAD2 decreased cell migration, invasion, and colony-forming abilities. Gene Set Enrichment Analysis (GSEA) indicated the involvement of OCIAD2 in oxidative phosphorylation (OXPHOS). Subsequently, mitochondrial metabolic assay demonstrated that OCIAD2 impairs OXPHOS function, accompanied by a metabolic shift toward glycolysis. These findings suggest that OCIAD2 promotes cancer progression through metabolic reprogramming, highlighting the role of OCIAD2 as a potential biomarker and therapeutic target for lung adenocarcinoma.

## Graphical abstract

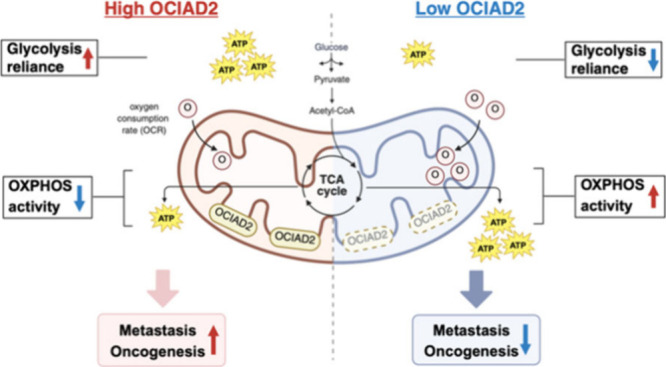

## Introduction

Following the successful completion of the Human Genome Project (HGP), the identification and functional annotation of cognate protein products have expanded rapidly. Nevertheless, approximately 30% of protein-coding genes lacked protein-level evidence in 2010, prompting the Human Proteome Organization (HUPO) to launch the global Human Proteome Project (HPP) to map the entire human proteome for a deeper understanding of human biological systems and disease.^1^ Notably, around 6.5% of protein-coding genes remain without direct protein-level evidence as of 2024, underscoring the importance of continuously completing this project.^2^ One international collaborative initiative of HPP is the Chromosome-centric HPP (C-HPP), which aims to identify and map all human protein sets in a chromosome-centric manner.^3^ A key mission of this initiative is to identify the uncharacterized protein existence level 1 (uPE1), which is devoid of any functional annotation in neXtProt, and missing proteins (MPs), which are defined as proteins that lack sufficient experimental data from mass spectrometry (MS) or other proteomic methods to qualify as protein existence level 1 (PE1), also need to be detected. Completing these investigations will not only lead to a more comprehensive understanding of the human proteome but also facilitate the discovery of novel cellular regulators, potential therapeutic targets, and biomarkers relevant to human biology and disease.^4^

Lung adenocarcinoma (LUAD), the predominant histologic subtype accounting for 50% of all lung cancer cases,^5^ has recently benefited from significant advances in early diagnosis and molecular therapeutics such as targeted therapy and immunotherapy.^6,7^ Nevertheless, its high incidence and limited 5-year survival rate remain critical challenges.^5,8^ Emerging evidence shows that characterizing the proteome to identify novel molecular biomarkers can improve clinical outcomes in LUAD,^9,10^ as proteins serve as the functional effectors of cells. Consequently, conducting proteomic studies of lung adenocarcinoma is essential to obtain a comprehensive view of the disease and enable the direct investigation of key effector proteins. Within the C-HPP, our research team is responsible for profiling proteins encoded by chromosome 4. In our previous study, the integrated proteogenomics analyses revealed the molecules associated with nonsmoking-related lung adenocarcinoma in East Asian populations,^11^ thereby providing insights into the pathogenesis, molecular mechanisms, etiology, and driving the discovery of potential druggable targets and clinical biomarkers for lung adenocarcinoma.

In this study, ovarian carcinoma immunoreactive antigen domain containing 2 (OCIAD2), an uPE1 protein encoded from chromosome 4, was identified as a dysregulated protein in lung adenocarcinoma according to our previous proteogenomic data sets.^11^ OCIAD2 was first identified in 2002 based on its high similarity to ovarian carcinoma immunoreactive antigen domain containing 1 (OCIAD1),^12^ both of which belong to the OCIA domain family. OCIAD1 was initially identified as an immunoreactive protein from the ovarian carcinoma cDNA expression library with ovarian cancer ascites using an immunoscreening approach.^13^ OCIAD1 has been reported to be overexpressed in metastatic and chemoresistant ovarian cancer cells, where OCIAD1 promotes cell adhesion to the extracellular matrix.^14,15^ Despite these insights into OCIAD1, the biological function of OCIAD2 remains poorly understood. Notably, the expression of OCIAD2 varies across different cancer types. Elevated levels of OCIAD2 protein have been observed in ovarian mucinous tumors^16^ and lung adenocarcinomas,^17,18^ whereas OCIAD2 expression significantly decreased in liver carcinomas,^19^ glioblastomas,^20^ and chronic lymphocytic leukemia.^21^ Given that the expression of OCIAD2 appears to play diverse roles in different cancers, further investigation is required to clarify its precise function, particularly its potential role in malignant tumor progression. A previous study has shown that elevated levels of OCIAD2 correlates with a poor prognosis^22^ in lung adenocarcinoma. However, the underlying molecular mechanisms remain unclear. Recently, a pathway enrichment analysis revealed that the OCIAD2 coexpressed genes derived from RNA-seq data sets are enriched in mitochondria-related signaling pathways.^23^ Given the modest correlation between RNA levels and expression of protein products,^24,25^ transcriptome-level analyses may not be sufficient to fully understand the role of OCIAD2 in lung adenocarcinoma. This highlights the critical need to examine the proteogenomic data of lung adenocarcinoma patients to elucidate the precise function of the invasive neuroblastoid OCIAD2.

To address this issue, the clinical relevance of OCIAD2 was unveiled by analyzing the clinical data obtained from public data sets and our prospective cohort of the Taiwan Cancer Moonshot (TwCM) Project.^11^ Several *in vitro* functional assays and Western blot analyses were conducted to investigate the impact of OCIAD2 on cellular processes. Furthermore, pathway enrichment analysis was conducted on the proteomic data derived from our cohort^11^ to determine the biofunctions of OCIAD2 and the OCIAD2-mediated signaling pathways in tumor initiation and progression. Furthermore, the mitochondrial metabolic change induced by the alteration of the OCIAD2 was measured to explore the functional role of the OCIAD2 in mitochondria-related pathways in lung adenocarcinoma. Overall, our study sheds light on the role of OCIAD2 in the cancer progression of lung adenocarcinoma.

## Materials and Methods

### Cell Culture and shRNA-Mediated Knockdown

A549, PC9, H1299, H1568, BEAS-2B, H1650, H1437, H3255, H1755, H1975, HCC827, and H838 cells were purchased from the American Type Culture Collection (ATCC). HOP-62, HOP-92, NCI-H23, NCI-H226, EKVX, NCI-H522, NCI-H322M, and NCI-H460 cells were purchased from the National Cancer Institute’s (NCI) Developmental Therapeutics Program (Bethesda, MD). PE-089, CL1–0, and CL1–5 were established from Taiwanese lung cancer patients.^26,27^ Cells were cultured in Roswell Park Memorial Institute Medium-1640 (RPMI-1640) medium supplemented with fetal bovine serum (FBS) and penicillin–streptomycin purchased from Life Technologies (Carlsbad, CA) at 37 °C under a 5% CO_2_ atmosphere. For this study, stable OCIAD2-silenced cell lines were established in A549 and NCI-H322 M cells using TRC1.Scramble (pLKO.1-shScramble; ASN0000000004) and two OCIAD2-shRNA-containing lentiviral vectors (pLKO_TRC005-shOCIAD2–1 and pLKO.1-shOCIAD2–2; TRCN0000318951 and TRCN0000129919), obtained from the National RNAi Core Facility (Academia Sinica, Taipei, Taiwan). On day 1, A549 and NCI-H322 M cells were seeded and cultured in the recommended medium at 37 °C in a 5% CO_2_ atm. The following day, cells were transfected with respective lentiviruses in Polybrene (8 μg/mL) containing medium. On day 3, fresh growth medium containing 2 μg/mL puromycin was added to select for puromycin-resistant clones.

### Real-Time Quantitative RT-PCR

We extracted RNA from the cultured cell lines using TRIzol reagent (Life Technologies, Carlsbad, CA), and one microgram of RNA was converted to cDNA using Superscript III reverse transcriptase (Life Technologies, Carlsbad, CA). cDNA was used as the template for the real-time quantitative RT-PCR on an ABI prism 7900 sequence detection system (Life Technologies, Carlsbad, CA). PCR reaction was set up as follows: 2 μL of cDNA, 0.3 μL of each primer (see below), and 5.0 μL of the PerfeCTa SYBR Green FastMix reagent (Quanta Bioscience, Gaithersburg, MD), adjusted to a total volume of 10 μL with nuclease-free water.

Primers used for SYBR Green assay in this study are OCIAD2 (forward 5′-GCA AGC AGA GCC TGT TGT TTT-3′; reverse 5′-CTA GTC CCT GGG TGA CAA GCA T-3′), TBP (forward 5′-CAC GAA CCA CGG CAC TGA TT-3′; reverse 5′-TTT TCT TGC TGC CAG TCT GGA C-3′).

The relative mRNA expression of target gene was normalized to an internal control, TATA-box binding protein (TBP), and the target/TBP mRNA ratio was calculated as 2^–ΔCT^ which ΔCT was determined as (CT_target_ – CT_TBP_). All data are the means from triplicate experiments.

### Cell Functional Assays

Since the cell proliferation, migration and invasion are common hallmarks of tumor cells and play important roles in tumorigenesis, several functional assays were conducted as previously described,^28,29^ including 3-[4,5-dimethylthiazole-2-yl]-2,5-diphenyltetrazolium bromide (MTT) assay, colony formation assay, and transwell migration and invasion assays. Briefly, 1000 cells were seeded into 96-well plate for 0, 24, 48, and 72 h in MTT assay and the absorbance at 570 nm was measured on a multiwell scanning spectrophotometer (Victor3; PerkinElmer, Boston, MA). Colony-forming ability was assessed by seeding 1000 cells into a 6-well plate, followed by methanol fixation and staining with 0.001% crystal violet for colonies counting after 1 week of incubation at 37 °C. For the transwell migration assay, 5000 cells suspended in serum-free medium were seeded onto the upside of a transwell (8 μm pore size; BD Biosciences, Franklin Lake, NJ) for 16–20 h and 750 μL medium containing 10% FBS was placed in the outer chamber. Subsequently, the cells migrating to the transwell outside were counted under a light microscope. For the transwell invasion assay, 50000 cells were seeded onto the upside of a transwell coated with Matrigel and the cells attached to the outside surface of the transwell were counted after 16–20 h.

### Western Blot Analysis

Whole cell lysates from cultured cell lines were first lysed in RIPA lysis buffer (25 mM Tris-HCl (pH7.6), 150 mM NaCl, 1% NP-40, 1% Sodium deoxycholate, and 0.1% SDS) containing protease and phosphatase inhibitor cocktails (Roche, Basel, Switzerland) and DTT (1:1000). To prepare mitochondrial and cytosolic fraction, the Mitochondria/Cytosol Fractionation Kit (BioVision, CA, USA) was used in accordance with the manufacturer’s protocol. Briefly, A549 cells were first collected by centrifugation and resuspended with 1X Cytosol Extraction Buffer Mix containing DTT and Protease Inhibitors. After incubation on ice for 10 min and thorough homogenization of cells, the sample was centrifuged at 700*g* for 10 min at 4 °C. Then, the supernatant was subjected to 10,000*g* centrifugation for 30 min at 4 °C. Subsequently, the given supernatant was saved as Cytosolic fraction and the pellet was resuspended by the Mitochondrial Extraction Buffer Mix for Mitochondrial fraction.

Samples were then loaded onto self-made 15% sodium dodecyl-sulfate polyacrylamide gel electrophoresis (SDS-PAGE) to separate the proteins and transferred onto PVDF membranes followed by appropriate antibodies as described below.

The OCIAD2 antibody (1:1000 – NBP2-98626, Novus Biologicals) was used to validate the OCIAD2 knockdown efficiency in A549 and NCI-H322 M shOCIAD2 cell lines. The mouse anti-MFN2 IgG (1:1000 – AB56889, Abcam) and the rabbit anti-HSP60 IgG (1:10,000 – 15282-1-AP, Proteintech) were used to detect the mitochondrial proteins. Anti-β-actin (GT5512, GeneTex) antibody was used as an internal control. After incubation with specific antibodies, the immunoblotting result was visualized by a chemiluminescence assay kit (Merck Millipore, Burlington, MA) and detected with the FUJIFILM LAS-3000 ECL system.

### ATP Production Assay

The Glycolysis/OXPHOS Assay Kit (Dojindo, Japan) was used to detect ATP production from glycolysis or oxidative phosphorylation in the control and OCIAD2 knockdown cell lines. The day before the assay, 5000 cells were seeded into each well of a 96-well microplate and cultured overnight in the 37 °C incubator with 5% CO_2_.

On the day of the assay, the cultured medium was replaced with 100 μL of fresh RPMI-1640 medium in the control group, while the other group was replaced with 100 μL of 22.5 mmol/L 2-deoxy-d-glucose (2-DG) solution. After five h of incubation at 37 °C in 5% CO_2_, 100 μL of ATP working solution containing luciferase was added to the cells in each well. The luminescence was measured using the SpectraMax iD3 microplate reader after incubation at 25 °C for 10 min. The total ATP production was determined from the control group, while glycolytic ATP production was calculated from the 2-DG-treated group. Oxidative ATP production was determined by subtracting glycolytic ATP from total ATP.

### Mitochondrial Respiration Measurement

To measure mitochondrial respiration function in control and OCIAD2 knockdown cell lines at dynamic aspects, the Cell Mito Stress assay was conducted on the Seahorse XFe24 Extracellular Flux Analyzer (Agilent, Santa Clara, CA, USA). The assay kit included three key reagents: oligomycin (an inhibitor of mitochondrial ATP synthase targeting ETC complex V), carbonyl cyanide *p*-(trifluoromethoxy) phenylhydrazone (FCCP) (a protonophore disrupting the mitochondrial inner membrane potential), and Rotenone/Antimycin A mix (a mixture that can shut down mitochondrial respiration by targeting ETC complex I and III, respectively). Before the Cell Mito Stress assay was performed, a titration test was recommended to identify the optimal dose of FCCP that yields a maximal oxygen consumption rate (OCR). According to the manufacturer’s instructions, FCCP concentrations ranging from 0.125 μM to 2.0 μM were tested in A549 and NCI-H322 M cell lines, and the results indicated that two cell lines are both sensitive to the FCCP when treated with 2.0 μM FCCP (Figure S5B–C). Thus, 2.0 μM FCCP was chosen as the optimum dosage for subsequent experiments.

On the day before the assay, adherent cells were seeded at 60000 cells/well in 100 μL 10% FBS RPMI-1640 media without any buffering reagents (HEPES and sodium bicarbonate) into the 24 well XFe24 cell culture plates and make sure the background wells (A1, B4, C3, and D6) contain no cells. Also, the sensor cartridge was hydrated with a calibrant filled in each well and was placed in a non-CO_2_ 37 °C incubator overnight to prevent evaporation of the calibrant. Thus, the probe tip of the sensor cartridge that detects the changes in the concentration of the O_2_ and pH can function correctly.

On the day of the assay, we replaced the 10% FBS RPMI-1640 media with the 2% FBS RPMI-1640 media and placed the plate in a non-CO_2_ 37 °C incubator for one h before the assay. Then, three kit components (oligomycin, FCCP, and Rotenone/Antimycin A mix) were prepared and loaded into the designated injector ports of the hydrated sensor cartridge. Following this preparation, the Seahorse Cell Mito Stress assay was conducted using the Seahorse XFe24 Extracellular Flux Analyzer. Upon completion of the assay, cells were washed three times with 2% FBS RPMI-1640 media, leaving 50 μL of medium in each well after the final wash. Subsequently, 50 μL trypan blue was added for staining and postassay cell counting. The oxygen consumption rate (OCR, pmol/min) was normalized to the cell number in each well and was presented as pmol/min per 1 × 10^3^ cells. All bioenergetic parameters were calculated according to the Seahorse XF Cell Mito Stress Test user guide: basal respiration was calculated by subtracting nonmitochondrial respiration from the last rate measurement before oligomycin injection; ATP-linked respiration was calculated by subtracting rate measurement after oligomycin injection from basal respiration; spare capacity was calculated as the increase in the level of the OCR following FCCP-induced uncoupling relative to basal respiration; nonmitochondrial respiration was measured after rotenone/antimycin A treatment; proton leak was measured by subtracting nonmitochondrial respiration from the rate after oligomycin treatment; maximal respiration was determined by subtracting nonmitochondrial respiration from the maximal OCR value after FCCP treatment. All parameter values were then expressed as percentages by setting the maximal OCR for each cell as 100%.

### Study Cohorts

The proteogenomic data was obtained from our previously established TwCM cohort described in a previous study.^11^ This study was approved by the Research Ethics Committees of Academia Sinica (approval number: AS-IRB02–106214). All patients included in this study provided written informed consent for sample collection, clinical data use, and related research applications. Patient samples were deidentified and assigned study identification numbers to protect participant confidentiality.

### Clinical Outcome Evaluation

To assess the clinical impact of OCIAD2 expression level, we first explore the correlation between OCIAD2 expression and survival outcomes by Kaplan–Meier Plotter (https://www.kmplot.com). Moreover, the clinical data of 89 LUAD patients from the TwCM cohort^11^ was analyzed, with patients stratified into high and low OCIAD2 groups using Log_2_ tumor-to-normal (T divided by N, abbreviated as T/N) expression ratio of 0.38, a cutoff considered indicative of significant dysregulation and used in our previous studies.^11,29^ The survival outcomes were calculated by the Kaplan–Meier method, and correlations between the OCIAD2 expression levels and 11 clinicopathological features were analyzed by the Chi-square test.

### Pathway Enrichment Analysis

To explore the cancer-related pathways associated with OCIAD2, the OCIAD2 coexpressed genes obtained from public RNA-sequencing data sets LinkedOmics (http://linkedomics.org/login.php)^30^ and cBioPortal (https://www.cbioportal.org)^31^ were initially derived from the TCGA-LUAD cohort (*n* = 515) and TCGA -LUAD cohort (*n* = 510) based on Pan-Cancer Atlas Studies, respectively. The OCIAD2 coexpressed genes were identified based on a Spearman correlation coefficient of *R* > 0.25 or *R* < −0.25. The differentially expressed gene related to OCIAD2 was then considered as *p* values < 0.05 (Welch’s *t* test). Subsequently, the differentially expressed gene associated with OCIAD2 was subjected to pathway enrichment analysis using Metacore software (GeneGo, MI, USA) and Ingenuity Pathway Analysis (IPA) (Qiagen, USA).

The proteomic data of patients from the TwCM cohort provided by our previous proteogenomics profiling^11^ is available in NCI Proteomics Data Commons (https://proteomic.datacommons.cancer.gov/pdc/) under accession numbers PDC000219 and PDC000220. 89 lung adenocarcinoma patients were first classified into high-OCIAD2 (Log_2_ T/N values ≥ 0.38, *n* = 67) and low-OCIAD2 (Log_2_ T/N values < 0.38, *n* = 22) groups. Then, the proteomic profile of two patient groups was subjected to Gene Set Enrichment Analysis (GSEA) and was analyzed in the gene set of GO:BP (Gene Ontology: biological process, 7647 gene sets) and CP:Reactome (Canonical Pathways: reactome, 1692 gene sets) by using GSEA software (Broad Institute, Cambridge, Massachusetts) (v 4.1.0).^32^ Additionally, 1031 differentially expressed proteins (*p* < 0.05 by Welch’s *t* test) between high-OCIAD2 (Log_2_ T/N values ≥ 0.38, *n* = 67) and low-OCIAD2 (Log_2_ T/N values < 0.38, *n* = 22) groups were identified from a total of 8526 proteins by Spearman correlation (*R* > 0.25 or *R* < −0.25). Subsequently, the 1031 proteins were analyzed by using Metacore software (GeneGo, MI, USA) to verify the results of public data sets.

### Metabolic Flux Score Estimation

To infer alterations in metabolic flux associated with OCIAD2 expression, proteomic data from high-OCIAD2 (Log_2_ T/N values ≥ 0.38, *n* = 67) and low-OCIAD2 (Log_2_ T/N values < 0.38, *n* = 22) patients in the TwCM cohort were analyzed using METAFlux (METAbolic Flux balance analysis) R package (v1.0.0).^33^ In particular, estimated flux scores were calculated for key mitochondrial bioenergetic pathways, including oxidative phosphorylation (Reaction ID: HMR_6921) and glycolysis (HMR_4358), among 1648 nutrient exchange (uptake/release) reactions as implemented by METAFlux. The resulting metabolic fluxes were then compared between patients with high-OCIAD2 and low-OCIAD2 expression levels.

### Independent Cohort Validation

Two independent large-scale proteomic data sets were utilized for validation analyses.^10,34^ Patients lacking the OCIAD2 expression data were excluded to avoid misinterpretation. In the first data set^10^ ranking was based on tumor-to-normal (T/N) OCIAD2 expression ratios, while in the second data set^34^ patients were ranked by the difference in OCIAD2 protein expression between paired tumor and adjacent normal tissues (T minus N). For both data sets, patients were stratified into high and low OCIAD2 groups using the median as the cutoff. Clinical outcome evaluation was performed on the first data set^10^ according to the methodology described in the Clinical Outcome Evaluation section. Pathway enrichment analysis was conducted on both data sets following the procedures outlined in the Pathway Enrichment Analysis section. Metabolic flux score estimation was calculated for both data sets using the parameters detailed in the Metabolic Flux Score Estimation section, with the ratios of T divided by N and 2^(T – N)^ values as respective inputs for the first^10^ and second^34^ data sets.

### Statistical Analysis

Statistical differences between the two groups are explained in the figure legends. All data in this study were presented using GraphPad Prism 9 (GraphPad Software, San Diego, CA). R Programming (version 4.3.1, Johns Hopkins University, Baltimore, MD) was used for advanced statistical data analysis.

## Results

### High OCIAD2 Expression Associated with Worse Survival in Lung Adenocarcinoma

According to our previous proteogenomics landscape of the Taiwan Cancer Moonshot cohort consisting of 89 lung adenocarcinoma patients in East Asia,^11^ the upregulation of OCIAD2 protein was observed in 95.5% (85/89) patients by comparing OCIAD2 level of tumor parts (T) with that in matched normal adjacent tissues (N), implying a strong correlation between high OCIAD2 expression level and the presence of lung adenocarcinoma (Figure 1A).

**Figure 1:**
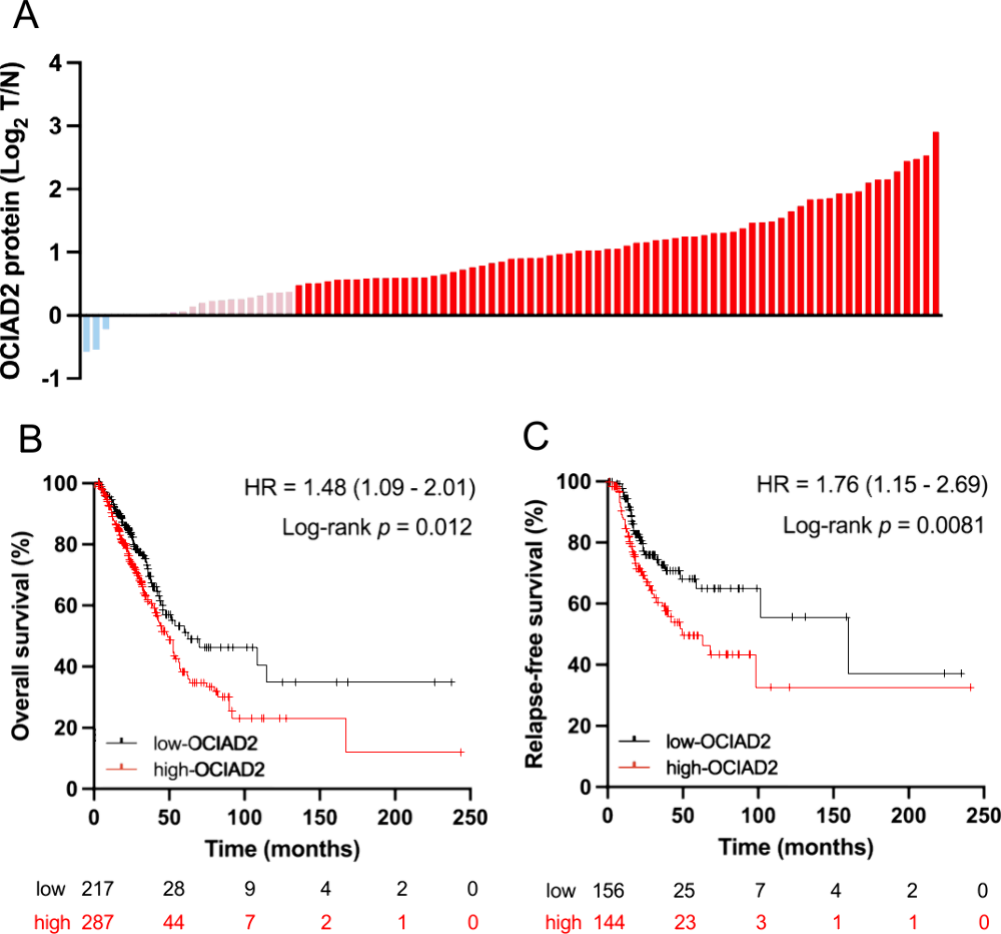
**Clinical relevance of OCIAD2 in lung adenocarcinoma**. (**A**) Relative expression levels of the OCIAD2 protein from tumors (T) and adjacent normal tissues (N) of 89 lung adenocarcinoma patients in the Log_2_ scale. Dark red: patients with Log_2_ T/N ratio ≥ 0.38. Light red: patients with 0.38 > Log_2_ T/N > 0. Light blue: patients with Log_2_ T/N ≤ 0. (**B** and **C**) Kaplan–Meier survival curves of overall survival (OS) (**B**) and relapse-free survival (RFS) (**C**) in lung adenocarcinoma patients grouped by high-OCIAD2 and low-OCIAD2 mRNA expression were analyzed using the public database. Red line = high-OCIAD2 mRNA group; black line = low-OCIAD2 mRNA group.

To further clarify the role of OCIAD2 expression in lung adenocarcinoma, the correlation between OCIAD2 expression and 11 clinicopathologic factors, including age, gender, smoking, differentiation, location, tumor size, lymph node involvement, pleural effusion, the mutational status of EGFR and TP53, and cancer staging, was performed by using the Chi-square test. Intriguingly, no significant association between the expression of OCIAD2 and these clinicopathologic features was observed (Table 1), suggesting that the correlation between the expression of OCIAD2 and lung adenocarcinoma may be independent of these factors. However, the independence might be due to the limited sample size.

**Table 1:** Clinicopathologic Characteristics of 89 Lung Adenocarcinoma Patients

**Characteristic**	**Total patients**	**Log**_**2**_ **T/N ≥ 0.38 Patient No. (%)**	**Log**_**2**_ **T/N < 0.38 Patient No. (%)**	***P*** **value** ^a^
**Patient No.**	*n* = 89	*n* = 67	*n* = 22	
**Age**				0.761
<60 years	30	22 (32.8%)	8 (36.4%)	
≥60 years	59	45 (67.2%)	14 (63.6%)	
**Gender**				0.860
Male	35	26 (38.8%)	9 (40.9%)	
Female	54	41 (61.2%)	13 (59.0%)	
**Smoking status**				0.341
Nonsmoker	77	56 (83.6%)	21 (95.5%)	
Ex-smoker	9	8 (11.9%)	1 (4.5%)	
Current smoker	3	3 (4.5%)	0 (0.0%)	
**Pathology differentiation** ^b^				0.851
Well	7	5 (7.6%)	2 (9.1%)	
Moderate	57	42 (63.6%)	15 (68.2%)	
Poor	24	19 (28.8%)	5 (22.7%)	
**EGFR status**				0.434
Wild-type	13	12 (17.9%)	1 (4.5%)	
L858R	35	25 (37.3%)	10 (45.5%)	
Exon 19 deletion	31	22 (32.8%)	9 (40.9%)	
Others	10	8 (12.0%)	2 (9.1%)	
**TP53 status**				0.337
Wild-type	60	47 (70.1%)	13 (59.1%)	
Mutations	29	20 (29.9%)	9 (40.9%)	
**Tumor size**				0.461
>2 cm	66	51 (76.1%)	15 (68.2%)	
≤2 cm	23	16 (23.9%)	7 (31.8%)	
**Visceral pleural status** ^c^				0.321
P0	69	53 (79.1%)	16 (73.0%)	
P1	10	9 (13.4%)	1 (4.5%)	
P2	6	3 (4.5%)	3 (14.0%)	
P3	2	1 (1.5%)	1 (4.5%)	
NA	2	1 (1.5%)	1 (4.5%)	
**Primary tumor location** ^d^				0.595
RUL	26	21 (31.3%)	5 (22.7%)	
LUL	22	14 (20.9%)	8 (36.4%)	
RML	6	5 (7.5%)	1 (4.5%)	
RLL	17	14 (20.9%)	3 (13.6%)	
LLL	18	13 (19.4%)	5 (22.7%)	
**Staging N**				0.686
N0	74	56 (83.6%)	18 (81.8%)	
N1	5	3 (4.5%)	2 (9.1%)	
N2	10	8 (11.9%)	2 (9.1%)	
**Stage**				0.303
IA	40	28 (41.8%)	12 (54.5%)	
IB	31	27 (40.3%)	4 (18.2%)	
II	6	4 (5.9%)	2 (9.1%)	
III–IV	12	8 (12.0%)	4 (18.2%)	

a Chi-square test.

b Some patients without information.

c P0: lack of pleural invasion beyond the elastic layer, P1: invasion beyond the elastic layer, P2: invasion to the surface of the visceral pleura, P3: invasion of the parietal pleura.

d RUL: right upper lobe, LUL: left upper lobe, RML: right middle lobe, RLL: right lower lobe; LLL: left lower lobe.

To evaluate the clinical impact of OCIAD2, the survival analysis was performed by Kaplan–Meier Plotter (https://www.kmplot.com, accessed on 2023/5/20)^35^ and the results showed that the high mRNA expression of OCIAD2 is significantly associated with poor overall survival (*p* = 0.012, Figure 1B) and relapse-free survival (*p* = 0.0081, Figure 1C) in the TCGA-LUAD cohort. Further survival analysis using proteomic data from our cohort was performed to evaluate the prognostic significance of OCIAD2. A series of cutoff values for Log_2_ T/N ranging from 0.38 to 1.4 were tested to minimize the overfitting effect, with 1.055 yielding the strongest stratification (Table S1). Using this optimized cutoff value, high OCIAD2 protein level was significantly associated with worse overall survival (*p* = 0.0097), though not with relapse-free survival (*p* = 0.12; Figure S1A). In addition, independent validation using a large-scale proteomic data set^10^ also demonstrated that high OCIAD2 expression was associated with worse overall survival, but not with disease-free survival (Figure S1B), further supporting our findings from the TwCM cohort proteomic data. These findings were also consistent with a previous report indicating that elevated OCIAD2 protein expression is significantly correlated with worse prognosis in lung adenocarcinoma.^22^

Taken together, our results highlighted a correlation between elevated OCIAD2 expression and worse clinical outcomes in lung adenocarcinoma, supported by both transcriptome- and protein-level evidence. Furthermore, the consistent observations in both our cohort and public data sets indicated that OCIAD2 might serve as a universal prognostic factor across different populations, emphasizing its value as an important biomarker in lung adenocarcinoma.

### OCIAD2 Promotes Lung Cancer Progression by Mediating Motility-Related Processes

To elucidate the molecular mechanisms underlying the oncogenic activity of OCIAD2, the role of OCIAD2 on cellular processes in lung adenocarcinoma was identified by analyzing the OCIAD2 coexpressed genes obtained from the public domain RNA-sequencing data sets LinkedOmics (http://linkedomics.org/login.php, accessed on 2023/5/20)^30^ and cBioPortal (https://www.cbioportal.org, accessed on 2023/5/20)^31^ using MetaCore pathway analysis software. The differentially expressed genes (DEGs) associated with OCIAD2 have been considered if the *p* value was <0.05 (Welch’s *t* test) and the Spearman correlation coefficient was *R* > 0.25 or *R* < −0.25, as the volcano plot shown (Figure 2A–B). From the LinkedOmics data set, there is a total of 1,611 OCIAD2-associated DEGs, consisting of 716 genes showing a positive correlation (red dots, named up-regulated DEGs) with OCIAD2 and 895 genes with a negative correlation (blue dots, named downregulated DEGs) (Figure 2A). There are 1,537 differentially expressed genes related to OCIAD2 in the cBioPortal data set, with 692 genes showing a positive correlation (red dots) and 845 genes showing a negative correlation (blue dots) (Figure 2B). There is an obvious overlap of up-regulated DEGs between the two data sets (70.67%, 583/825) (Figure S2A), which is also observed in downregulated DEGs (65.40%, 688/1,052) (Figure S2B).

**Figure 2:**
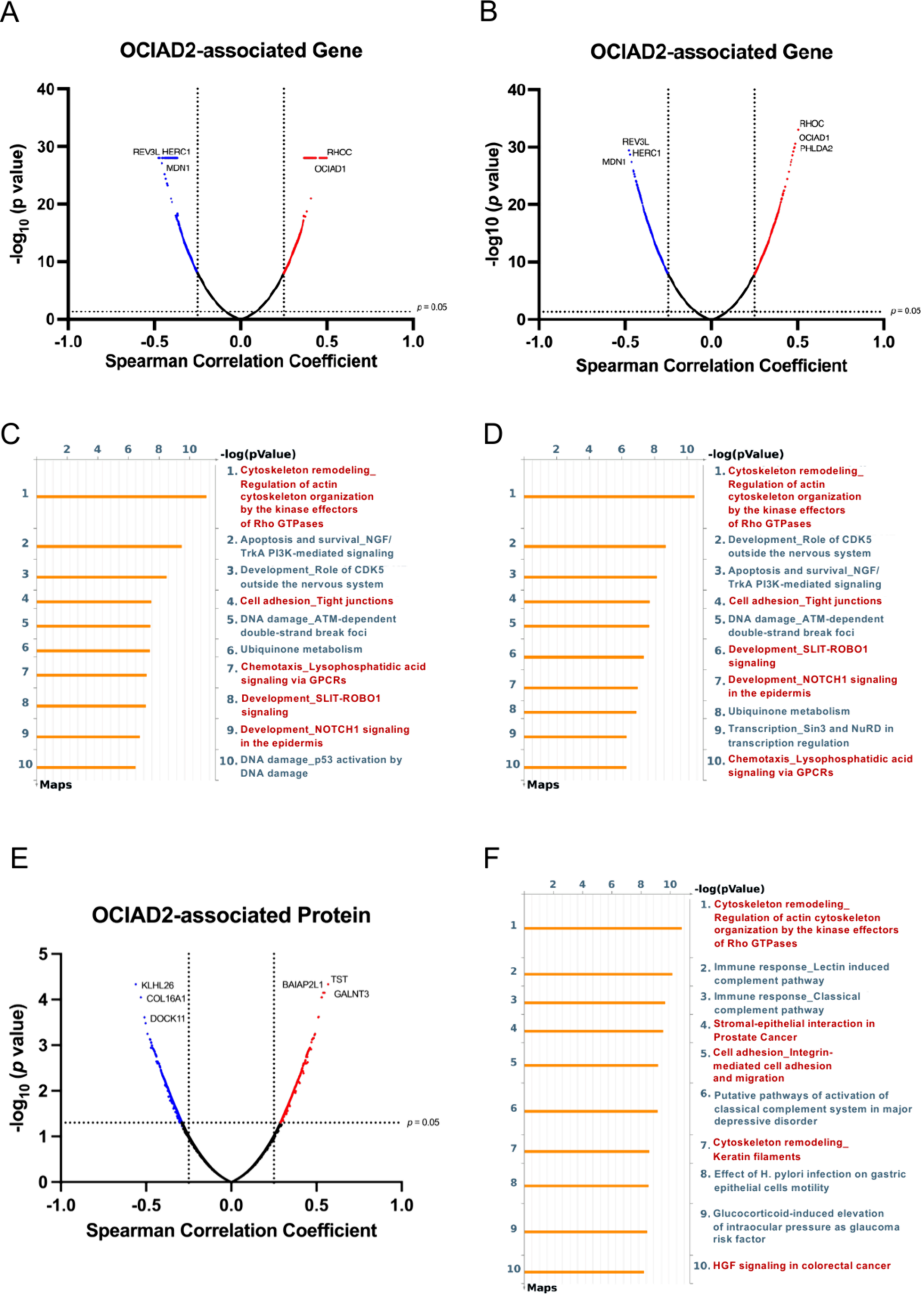
**Pathway analysis of OCIAD2-associated in lung adenocarcinoma progression**. (**A** and **B**) Volcano plots display the coexpressed genes of OCIAD2 in LUAD obtained from LinkedOmics (**A**) and cBioPortal (**B**) database which identified with a *p* value <0.05 by Welch’s *t* test (*y*-axis) and | Spearman correlation coefficient (R) | (*x*-axis) > 0.25. The positively correlated genes with *R* > 0.25 (*x*-axis) are in red and the negatively correlated genes with *R* < −0.25 (*x*-axis) are in blue. (**C** and **D**) The top 10 enriched pathways were analyzed by Metacore using the data assessed from LinkedOmics (**C**) and cBioPortal (**D**) database. Cell motility-related pathways were highlighted in red. (**E**) Differentially expressed proteins derived from Taiwan Cancer Moonshot cohort were identified with a *p* value < 0.05 by Welch’s *t* test (*y*-axis) and | Spearman correlation coefficient (R) | (*x*-axis) > 0.25. Of the 1031 differentially expressed proteins, 508 up-regulated proteins were colored red, while 523 downregulated proteins were marked blue in the volcano plot. (**F**) The top 10 enriched pathways were analyzed using the Taiwan Cancer Moonshot cohort data with Metacore. Red-colored pathways were associated with cell motility.

Metacore pathway analysis of OCIAD2-associated DEGs derived from two public data sets indicated enrichment in various aspects of cell movement, including cytoskeleton remodeling and cell adhesion, both of which are known to play important roles in cancer progression (Figure 2C–D). To further validate these findings, a total of 8,526 proteins derived from the TwCM cohort were analyzed and filtered by Spearman correlation (*R* > 0.25 or *R* < −0.25). Subsequently, 1031 differentially expressed proteins between high-OCIAD2 (Log_2_ T/N values ≥ 0.38, *n* = 67) and low-OCIAD2 (Log_2_ T/N values < 0.38, *n* = 22) groups were identified by Welch’s *t* test based on the cutoff value of *p* < 0.05. Among the 1,031 OCIAD2-associated proteins, 508 proteins (red dots) showed a positive correlation with OCIAD2, while 523 proteins (blue dots) showed a negative correlation (Figure 2E). Further analysis of these 1,031 differentially expressed proteins using Metacore revealed that three of the top 10 ranked pathways are associated with cell movement (Figure 2F), aligning with the RNA-level results from public databases (Figure 2C–D). The concordance of findings at both the RNA and protein levels emphasized that the presence of OCIAD2 might be a critical factor influencing cell motility pathways in lung adenocarcinoma across diverse omics analyses.

According to the pathway analysis, OCIAD2 might be involved in cancer cell motility. To further validate the functional role of OCIAD2 *in vitro*, stably circulating OCIAD2-silenced cell clones were established. First, the mRNA expression levels of OCIAD2 in 23 lung cancer cell lines were measured by reverse transcription quantitative real-time polymerase chain reaction (RT-qPCR) (Figure S3A). The low-expressing cell line A549 and the high-expressing cell line, NCI-H322M, were identified for further OCIAD2 knockdown experiments. OCIAD2 was silenced through lentiviral transfection of three independent shOCIAD2 viruses in A549 cells, and the knockdown efficiency was validated at both mRNA and protein levels using RT-qPCR and Western blotting (Figure 3A).

**Figure 3:**
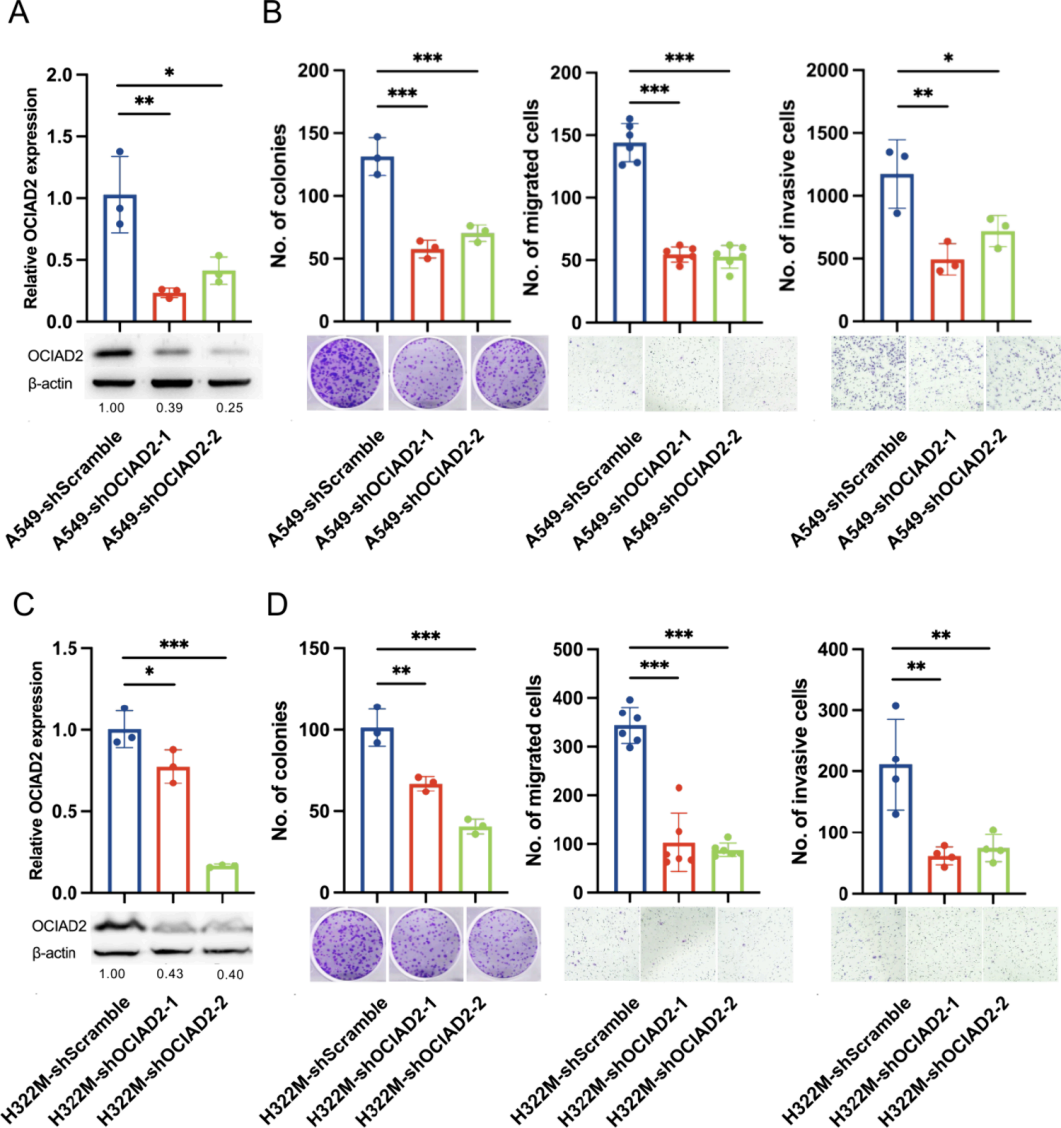
**Functional assessment of OCIAD2 in lung cancer cells**. (**A** and **C**) OCIAD2 in A549 (**A**) and NCI-H322 M (**C**) cells was evaluated through real-time quantitative RT-PCR (mean ± SD, *n* = 3), using TATA-box binding protein (TBP) as the internal control. Western blotting revealed the OCIAD2 protein expression level in shScramble control cells and two OCIAD2-silenced cells (shOCIAD2–1 and shOCIAD2–2). Numbers below the Western blot images represent the levels of expression of the β-actin for OCIAD2 quantified by ImageJ and normalized to β-actin as a loading control. (**B** and **D**) Three functional assays were conducted on A549 (**B**) and NCI-H322 M (**D**) cells, including colony formation assay (left), transwell migration assay (middle), and transwell invasion assay (right). All data are presented as the mean ± SD. Statistical significance was determined as *p < 0.05, **p < 0.01, and ***p < 0.001 by one-way ANOVA and Tukey’s multiple comparisons tests, compared to the shScramble control cells.

Subsequently, various cell functional assays, including MTT assay, colony formation assay, and transwell migration and invasion assays, were performed on OCIAD2-silenced and shScramble control A549 cells to characterize the phenotypic changes. Functional assays revealed that the knockdown of the OCIAD2 significantly decreased colony formation, migration, and invasion abilities in A549 cells (Figure 3B). In contrast, MTT assays indicated that OCIAD2 knockdown did not significantly influence cell proliferation at various time points (Figure S3B).

To validate whether the effects of OCIAD2 knockdown on cell functions are universal in lung cancer cells, the functional assays were conducted in NCI-H322 M cells, which exhibit higher mRNA expression of OCIAD2 (Figure S3A). OCIAD2-silenced and control cell clones of NCI-H322 M were established through lentiviral transfection of three independent shOCIAD2 constructs, followed by validation of knockdown efficiency (Figure 3C). OCIAD2-silenced NCI-H322 M cells similarly showed decreased colony formation, migration, and invasion abilities (Figure 3D) without short-term cell proliferation changes (Figure S3C). Taken together, the cellular functional assays demonstrated that the downregulation of the cellular component of OCIAD2 inhibits motility, invasion, and colony formation of lung cancer cells. The results supported the idea that OCIAD2 may promote cancer progression by modulating the process of cellular movement and invasion.

### OCIAD2 Suppresses OXPHOS Activity while Promoting Glycolytic Adaptation

Our findings in Figure 3 demonstrated that elevated levels of OCIAD2 expression might promote cancer progression in lung adenocarcinoma via cell motility-related processes; however, the underlying mechanisms of the OCIAD2-mediated signaling pathways remained unclear. Recent studies have suggested that OCIAD2 is involved in several mitochondrial functions, especially in mitochondrial bioenergetics.^23,36^ Consistent with these observations, the OCIAD2 protein was found mostly localized to the mitochondria in A549 cells by Western blotting, with its presence in the mitochondrial fraction and the absence in the cytosolic fraction (Figure 4A). Additionally, the expression level of Mitofusin-2 (MFN2) protein, a mitochondrial membrane protein involved in mitochondrial fusion, was significantly elevated in the cells of the OCIAD2-silenced group compared to the control cells, suggesting a potential association between OCIAD2 and mitochondrial function of the OCIAD2 complex (Figure 4B). Aligned with the *in vitro* findings, the proteomic data of the TwCM cohort (Taiwan Cancer Proteogenomic Knowledgebase: https://www.twcms.org/moonshot/portal/index) revealed a trend toward a negative correlation between OCIAD2 and MFN2 protein expression levels in lung adenocarcinoma patients, although this correlation was not statistically significant (Figure S4A).

**Figure 4:**
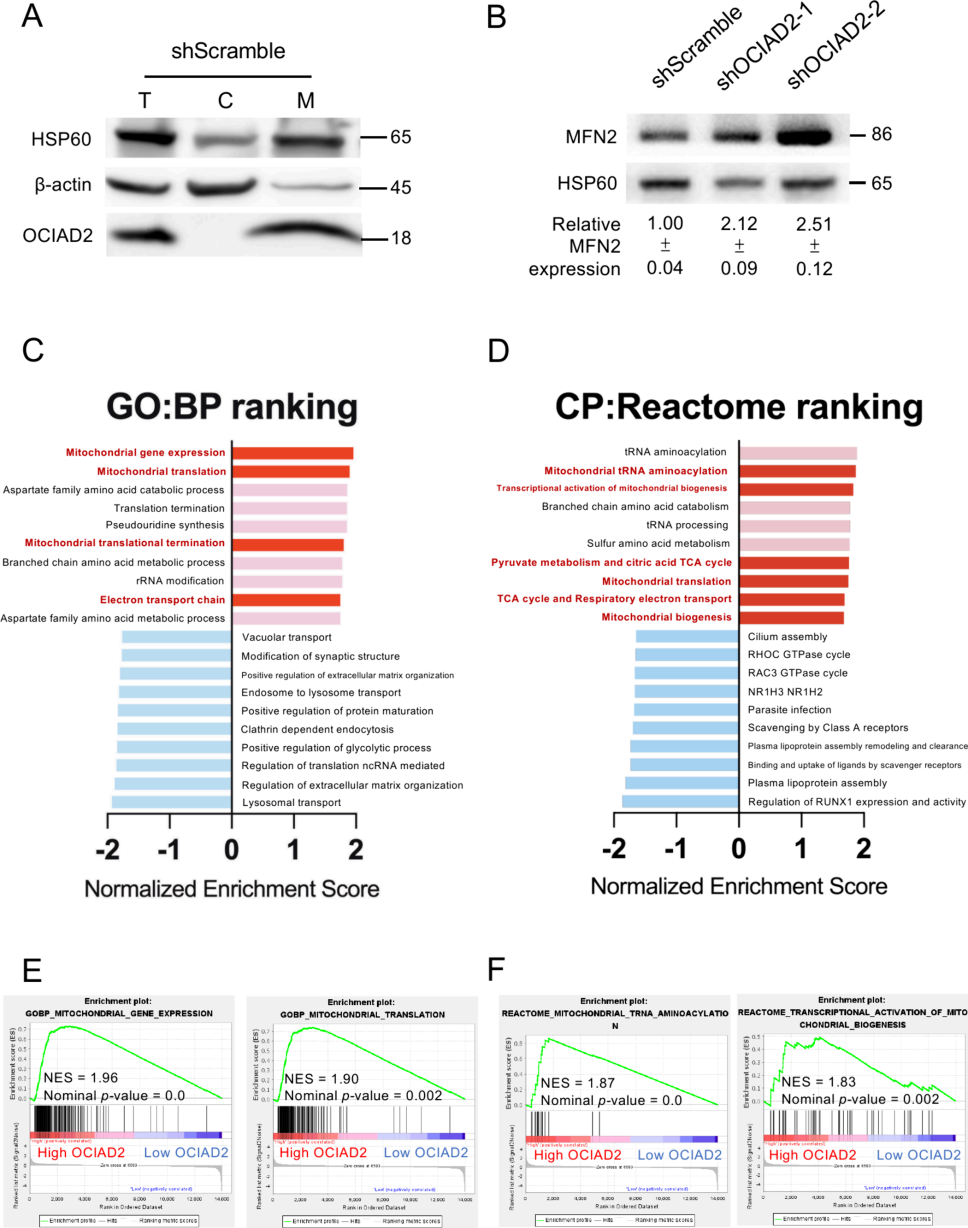
**Investigation of the role of OCIAD2 in mitochondria-associated pathways in lung cancer cells**. (**A**) The OCIAD2 protein in cytosolic (C) and mitochondrial (M) fractions extracted from A549-shScramble cells was detected by Western blotting. The fractionations of mitochondria and cytosol were validated by the specific markers HSP60 (heat shock protein 60) and β-actin, respectively. T: total cell lysate; C: cytosolic fraction; M: mitochondrial fraction. (**B**) Western blot analysis was performed on mitochondrial fractions in OCIAD2-silenced and scramble control A549 cells to determine the expression level of Mitofusin-2 (MFN2) protein. The relative expression of MFN2 was quantified by ImageJ software and normalized to mitochondrial loading control HSP60 (mean ± SD, *n* = 3). (**C** and **D**) Normalized Enrichment Score (NES) of the top 10 positively (red) and negatively (blue) enriched gene sets resulted from GSEA analysis of high-OCIAD2 group (Log_2_ T/N ≥ 0.38) vs low-OCIAD2 group (Log_2_ T/*N* < 0.38). Of note, the mitochondrial-related pathways are shown in dark red columns. (**E** and **F**) Enrichment plots of 2 top-ranked pathways enriched in (**C**) GO-BP and (**D**) CP-Reactome gene sets show the normalized enrichment score (NES) and ranking metric score.

To further investigate the signaling pathways, particularly the mitochondrial pathways, affected by OCIAD2 in lung adenocarcinoma, the proteomic data from 89 lung adenocarcinoma patients in Taiwan Cancer Moonshot cohort was first divided into high-OCIAD2 group (Log_2_ T/N ≥ 0.38) and low-OCIAD2 group (Log_2_ T/N < 0.38). Subsequently, the proteomic data was subjected to GSEA analysis. As expected, pathway enrichment analysis of GO:BP (Gene Ontology: Biological Process) and CP:Reactome (Canonical Pathways: Reactome) gene sets revealed that the high-OCIAD2 group was enriched in several mitochondrial functions, including mitochondrial gene expression (NES = 1.96), mitochondrial translation (NES = 1.90), mitochondrial tRNA aminoacylation (NES = 1.87), and transcriptional activation of mitochondrial biogenesis (NES = 1.83) (Figure 4C–F). These findings were validated through further analyses using independent large-scale proteomic data sets.^10,34^ Both data sets showed consistent enrichment of mitochondria-related pathways in patients with high OCIAD2 expression (Figure S4B–C). Collectively, results from our TwCM cohort (Figure 4C–F), the independent proteomic data sets (Figure S4B–C), and RNA-seq data from the TCGA-LUAD cohort (Figure S4D) highlight a potential role for OCIAD2 in regulating mitochondrial metabolic functions, including the electron transport chain (ETC), TCA cycle, and oxidative phosphorylation (OXPHOS). Given that the deregulation of cellular energetics is a key hallmark of cancer,^37^ it is essential to further explore the functional impact of OCIAD2 on mitochondrial bioenergetics in lung cancer cells.

In the Cell Mito Stress Test using the Seahorse XFe24 Extracellular Flux Analyzer, the activity of mitochondrial oxidative phosphorylation (OXPHOS) can be assessed by measuring the oxygen consumption rate (OCR) in living cells. Real-time measurements of OCR in A549 and NCI-H322 M cells are shown as kinetic profiles (Figure 5A–B), and the bioenergetics parameter values were calculated based on the assay profiles (Figure S5A) and normalized to cell number. Key assay parameters were displayed as bar charts (Figure 5C–E and Figure S5D). It was noted that the concentration of OCIAD2-silenced cells exhibited significantly higher basal respiration and ATP-linked respiration compared to shScramble control cells in both A549 and NCI-H322 M cells (Figure 5C–D). Spare respiratory capacity was significantly higher in A549-shOCIAD2–1, A549-shOCIAD2–2, and H322M-shOCIAD2–2 cells (Figure 5E). Taken together, the Seahorse metabolic flux analysis revealed that shScramble control cells, which have higher levels of expression of the OCIAD2 gene, showed decreased levels of the corresponding OXPHOS activity compared to those of the OCIAD2-silenced cells, particularly in A549 cells.

**Figure 5:**
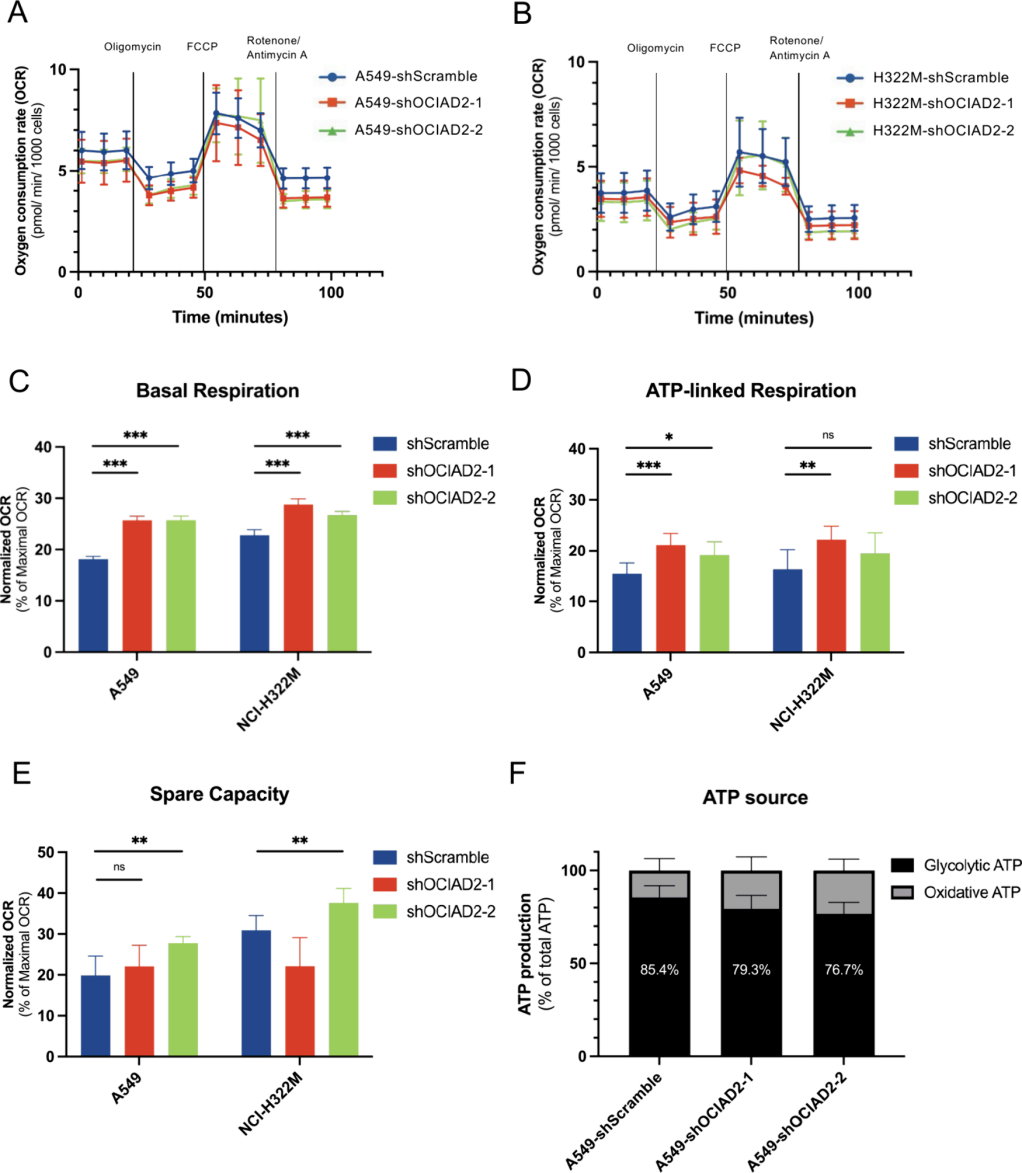
**Influence of OCIAD2 on mitochondrial metabolic functions in lung cancer cells**. (**A** and **B**) The oxygen consumption rate (OCR) was measured in real-time in A549 (**A**) and NCI-H322 M (**B**) cells transduced with shScramble or shOCIAD2 constructs following sequential injections of Oligomycin, FCCP, and Rotenone/Antimycin A. (**C–E**) Quantification of Basal respiration (**C**), ATP-linked respiration (**D**), and Spare capacity (**E**) in the A549 and NCI-H322 M cells. Data in (A-E) represent the mean ± SD (*n* = 9, 3 technical replicates × 3 biological replicates). Statistical significance for (**C**–**E**) was determined as *p < 0.05, **p < 0.01, and *** *p* < 0.001 by one-way ANOVA and Tukey’s multiple comparisons tests, compared to the shScramble control cells. (**F**) The relative luminescence unit (RLU) values were used to determine the proportional contributions of ATP generated by glycolysis and OXPHOS in A549 shScramble and A549 shOCIAD2 cells. Glycolytic ATP was indicated by the decreased ATP level under 2-DG treatment, while oxidative ATP was calculated by subtracting glycolytic ATP from the total ATP (represented by ATP levels in the nontreated group). All data were displayed as the mean ± SD (*n* = 3) and normalized to the total ATP.

Given that cancer cells with impaired OXPHOS function commonly exhibit glycolytic adaptation, known as the Warburg effect, to meet increased energy demands, we measured ATP production from the glycolysis of OXPHOS and glycolysis using the glycolysis/OXPHOS assay kit to examine the interplay between these two pathways. A549 cells were selected for this experiment as they showed a more pronounced reduction in OXPHOS activity than NCI-H322 M cells, as indicated by our Seahorse metabolic flux analysis (Figure 5A–E). The control group received no treatment, while the glycolytic group was treated with 2-deoxy-d-glucose (2-DG) to inhibit ATP synthesis by glycolysis and the OXPHOS group was inferred by subtracting the reduced ATP level observed under 2-DG treatment from total ATP measured in the untreated control. The results indicated that glycolytic ATP production constitutes a smaller proportion of total ATP in OCIAD2-silenced cells (79.3% and 76.7%) compared to shScramble control cells (85.4%) (Figure 5F), indicating that lung cancer cells with higher OCIAD2 expression displayed increased dependence on glycolytic ATP production. To further validate our functional findings, we conducted METAFlux analysis using the TwCM proteomic cohort and two independent large-scale proteomic data sets.^10,34^ In the TwCM cohort, METAFlux results were consistent with our experimental data, showing that patients with high OCIAD2 expression exhibited reduced OXPHOS activity and increased glycolytic flux (Figure S5E). Analysis of the two additional data sets^10,34^ yielded partially consistent trends (Figure S5F–G). Collectively, these findings demonstrated that elevated levels of OCIAD2 expression contribute to metabolic reprogramming toward glycolytic dependence in lung adenocarcinoma.

## Discussion

In this study, OCIAD2 was identified as an overexpressed protein in tumor tissues of lung adenocarcinoma patients, based on proteogenomics data from the TwCM cohort.^11^ The clinical relevance showed that elevated levels of OCIAD2 correlated with prolonged overall and relapse-free survivals. Additionally, the cellular functional assays unveiled that the knockdown of the OCIAD2 inhibits cancerous phenotypes including colony-forming, migration, and invasion activities. Mitochondrial metabolic assays were further performed based on the findings from the enrichment analysis, which indicated that OCIAD2 is involved in mitochondria-related pathways. The metabolic assays revealed that OCIAD2 suppresses OXPHOS activity, while promoting reliance on glycolysis. Taken together, our findings suggest that OCIAD2 may promote lung adenocarcinoma progression through metabolic reprogramming.

A previous study has reported a significant correlation between high OCIAD2 protein expression and worse overall survival in lung adenocarcinoma assessed by IHC staining assays,^22^ which is consistent with our results of survival analysis using a public data set (OS: *p* = 0.012, RFS: *p* = 0.0081) (Figure 1B–C). Our study further evaluated the clinical relevance of OCIAD2 in lung adenocarcinoma patients within the TwCM cohort, which features comprehensive proteogenomic profiles and clinical data. This approach helped minimize the potential bias arising from incomplete clinicopathological information that may not be fully clarified in public databases. The results showed that high OCIAD2 protein expression is significantly associated with worse overall survival (*p* = 0.0097), whereas the hazard ratio (1.72) of relapse-free survival suggested a potential trend toward worse outcomes (*p* = 0.12; Figure S1A). It is noted that no significant difference observed in relapse-free survival might be attributable to the limited follow-up period, highlighting the need for longer follow-up monitoring. Furthermore, other clinical information such as age, gender, smoking, differentiation, location, tumor size, lymph node involvement, pleural effusion, mutational status of EGFR and TP53, and cancer staging in our cohort were also analyzed. No significant correlation between OCIAD2 expression and 11 clinicopathologic factors was observed, implying that OCIAD2 might serve as an independent prognostic factor in lung adenocarcinoma (Table 1). It is worth noting that a previous immunohistochemistry-based study reported significant associations between high levels of OCIAD2 and factors, such as T factor of TNM staging (*p* = 0.0024) and pathological stage (*p* = 0.0003).^22^ However, the OCIAD2 expression detected by the summed percentage of positively stained cells in the previous study may not provide a quantitative measurement for precisely dividing patients into high- and low-OCIAD2 groups. Consequently, patients in the high-OCIAD2 group may have already undergone more extensive malignant transformation, thus exhibiting additional cancerous phenotypes. In contrast, our study divided patients into two groups using proteomic data with the defined cutoff point of protein expression Log_2_ T/N ≥ 0.38, allowing for more refined stratification. This may partly explain the absence of correlation with established clinicopathologic factors in our cohort and further support the potential role of OCIAD2 as an independent prognostic biomarker in lung adenocarcinoma.

A recent study has suggested the oncogenic function of OCIAD2 in tumor progression, as reduced cell proliferation and migration were observed in HEK293 cells, a human embryonic kidney cell line, with OCIAD2 silencing.^38^ The impact of OCIAD2 on cell movement was later confirmed by the wound healing assay in A549 and HCC827 lung cancer cells.^23^ Our study validated the upregulation of OCIAD2 in the process of cell motility by analyzing proteogenomics data obtained from our cohort using MetaCore software (Figure 2). Additionally, several cell functional assays were conducted to characterize the phenotypic changes in lung cancer cells given the OCIAD2 shRNA treatment, including MTT assay, colony formation assay, transwell migration, and invasion assays. Although the MTT assay showed no significant differences between shScramble control and shOCIAD2 cells (Figure S3B–C), colony formation was markedly reduced in OCIAD2 knockdown cells, indicating that tumorigenesis is obviously suppressed by OCIAD2 knockdown. Furthermore, our results showed that the silencing of the peptide in the cytosolic RNA complex of OCIAD2 inhibits cell migration and invasion abilities in both A549 (Figure 3B) and NCI-H322 M cell lines (Figure 3D), providing a more comprehensive understanding of the cancerous phenotypes associated with OCIAD2 in lung adenocarcinoma.

Previous study has implicated OCIAD2 in mitochondrial function using various experimental approaches.^23^ Consistent with these findings, our Western blot analysis confirmed that the expression of OCIAD2 is predominantly localized to mitochondria (Figure 4A). In addition, prior GSEA results based on RNA-seq data from public databases also demonstrated enrichment of OCIAD2 in mitochondria-related pathways in lung adenocarcinoma, supporting a role for OCIAD2 in mitochondrial regulation.^23^ Additionally, the immunopurification followed by quantitative mass spectrometry conducted in HEK293 cells indicated that OCIAD2 interacts with the electron transport chain (ETC) complexes.^36^ In this study, we further analyzed the proteomic data of our cohort to determine the enriched pathways of the OCIAD2 in lung adenocarcinoma. This approach provides more direct and deeper mechanistic insights, complementing the results that used transcriptomic data from public databases or proteomic data from cell lines. Our results from GSEA and IPA indicated that high expression levels of OCIAD2 are correlated with various mitochondrial metabolic functions (Figure 4C–F and Figure S4B–D), aligning with previous reports. Then, Seahorse metabolic flux analysis enabling dynamic metabolic profiling showed that lung cancer cells with higher OCIAD2 expression levels exhibited reduced OXPHOS activity, especially in A549 cells (Figure 5C–E). Furthermore, enhanced reliance on glycolytic ATP production was observed in A549 shScramble cells (Figure 5F), suggesting that elevated OCIAD2 expression might lead to impaired OXPHOS function but increased glycolytic activity. Computational validation using METAFlux analysis across three independent clinical proteomic cohorts provided partial support for these findings (Figure S5E–G). This shift toward glycolytic metabolism in tumors with high levels of expression of the ligand of OCIAD2 may act as a compensatory mechanism to sustain energy production under mitochondrial dysfunction, facilitating rapid ATP generation and contributing to tumor progression. This phenomenon reflects the well-known “Warburg effect,” a hallmark of cancer metabolism.^37^

Mitofusin-2 (MFN2) is a mitochondrial protein involved in mitochondrial fusion, bioenergetics, and autophagy.^39^ Overexpression of *Mfn2* has been reported to inhibit cell proliferation, attributed to the Mfn2-dependent inhibition of the Ras-Raf-ERK signaling pathway.^40^ In our study, OCIAD2 knockdown cells with increased MFN2 protein expression (Figure 4B) exhibited decreased cell motility, including migration, invasion, and colony-forming abilities (Figure 3). Moreover, a previous study demonstrated that Mfn2 deficiency in rats inhibited mitochondrial bioenergetics, particularly through repression of ETC complexes involved in OXPHOS.^41^ This observation aligns with our findings that OCIAD2 shScramble cells with reduced MFN2 protein levels (Figure 4B) showed decreased OXPHOS activity (Figure 5). Furthermore, given that MFN2 is crucial for mitochondrial fusion, its reduction has been reported to disrupt mitochondrial morphology and function, including suppression of electron transport chain (ETC) complexes required for OXPHOS, consequently forcing cancer cells to rely more heavily on glycolytic ATP production.^41–43^ While previous research suggested that OCIAD2 maintains mitochondrial integrity,^23^ our data indicated that the relationship between OCIAD2 and mitochondrial integrity might be more complex. Specifically, we observed that elevated OCIAD2 expression correlates with reduced MFN2 protein levels (Figure 4B), which might compromise mitochondrial integrity through impaired fusion processes, as evidenced by decreased OXPHOS activity and compensatory glycolytic reliance (Figure 5).

To sum up, our study proposes that elevated levels of the level of the expression of the OCIAD2 might attenuate the level of the OXPHOS function accompanied by compensatory activation of glycolysis, contributing to cancer progression in lung adenocarcinoma. Intriguingly, a previous study hypothesized that OCIAD2 enhances the survival and motility of lung cancer cells by inhibiting mitochondria-initiated apoptosis.^23^ Notably, several studies have demonstrated that OXPHOS deficiency is associated with an attenuation of apoptosis in cancer cells, as the mitochondrial electron transport chain (ETC) function and the mitochondrial Ca^2+^ uptake are essential for mitochondria-initiated apoptosis induction.^44,45^ Moreover, enhanced glucose metabolism resulting from OXPHOS deficiency is associated with increased apoptotic resistance in cancer cells, as glutathione generated from glucose metabolism inhibits the release of cytochrome c from the mitochondria, which is a crucial process involved in apoptotic pathway.^46^ Integrating these observations with our results, two hypotheses were proposed. First, the upregulation of OCIAD2 associated with decreased MFN2 might suppress the effect of OXPHOS and impair other mitochondrial functions involved in apoptotic pathways. This disruption might simultaneously induce compensatory glycolysis while weakening mitochondria-initiated apoptosis, thereby promoting cancer progression. Second, elevated levels of OCIAD2 expression coupled with MFN2 downregulation might cause an OXPHOS deficiency, resulting in an increased reliance on glucose metabolism. The increased production of glycolytic metabolites inhibits apoptotic processes through the redox inactivation of cytochrome c, thus offering cancer cells for enhanced apoptotic resistance. Although further investigation is still needed, these results suggest the intricate and interconnected networks within mitochondrial functions, highlighting the potential multifaceted oncogenic role of OCIAD2 in lung adenocarcinoma and underscoring the need for further exploration of its detailed molecular mechanisms.

## Conclusions

According to our previous proteogenomic study of the Taiwan Cancer Moonshot cohort, OCIAD2 was identified as highly expressed in patients with lung adenocarcinoma and strongly associated with unfavorable prognosis. MetaCore analysis of the proteomic data from the same cohort indicated that OCIAD2 promotes cancer progression, which was further validated through *in vitro* functional assays, including transwell migration, invasion, and colony formation assays. Additionally, the OCIAD2-mediated signaling pathways involved in lung adenocarcinoma were found to be related to several mitochondrial functions by GSEA analysis. Mitochondrial metabolic assay revealed that enhanced OCIAD2 expression inhibits OXPHOS activity while increasing glycolysis reliance.

Overall, our study proposed a novel oncogenic role for the poorly characterized protein, OCIAD2, suggesting its involvement in cancer progression through the regulation of metabolic reprogramming between the OXPHOS and glycolysis pathways in lung adenocarcinoma. These findings provide valuable insights into the underlying mechanisms mediated by OCIAD2 and highlight its potential as a negative prognostic biomarker in lung adenocarcinoma.

## Supplementary Material



## Data Availability

The proteomic data of Taiwan Cancer Moonshot (TwCM) cohort patients used and analyzed in this study are available in NCI Proteomics Data Commons (https://proteomic.datacommons.cancer.gov/pdc/) under accession numbers PDC000219 and PDC000220.

## Notes

Figure S1. Survival analysis of Taiwan Cancer Moonshot cohort and an independent large-scale proteomic data set; Figure S2. Overlap of differentially expressed genes identified in public data sets; Figure S3. Quantification and functional assessment of OCIAD2 in lung cancer cells; Figure S4. Exploring the impact of OCIAD2 on mitochondrial pathways; Figure S5. Optimization of the Seahorse Cell Mito Stress Test and normalization of all respiratory parameters; Figure S6. Whole membrane images of the original Western blots; Table S1. Prognostic capability of OCIAD2 on the overall survival of 89 lung adenocarcinoma patients (PDF)

## Abbreviations

ANOVA: Analysis of variance
ATCC: American Type Culture Collection
cDNA: Complementary DNA
C-HPP: Chromosome-centric Human Proteome Project
FBS: Fetal bovine serum
FCCP: carbonyl cyanide *p*-(trifluoromethoxy) phenylhydrazone
GO: Gene Ontology
GSEA: Gene set enrichment analysis
HPP: Human Proteome Project
IPA: Ingenuity Pathway Analysis
MPs: Missing proteins
MTT: 3-[4,5-Dimethylthiazole-2-yl]-2,5-diphenyltetrazolium bromide
OCIAD2: Ovarian carcinoma immunoreactive antigen domain containing 2
RT-qPCR: Reverse transcription quantitative real-time polymerase chain reaction
RPMI: Roswell Park Memorial Institute Medium
SDS-PAGE: Sodium dodecyl-sulfate polyacrylamide gel electrophoresis
shRNA: Short hairpin RNA
TwCM: Taiwan Cancer Moonshot
T/N: Tumor part compared with paired adjacent normal tissues
TBP: TATA-box binding protein
TCGA: The Cancer Genome Atlas
uPE1: Uncharacterized protein existence level 1
