# Supplementary material for: OCIAD2 Promotes Cancer Progression via Metabolic Reprogramming in Lung Adenocarcinoma
Source: J Proteome Res. 2025 Jul 21;24(8):4139–53. doi: 10.1021/acs.jproteome.5c00273 (PMC12323000; doi:10.1021/acs.jproteome.5c00273)
Supplement: Supplementary file 1 [file pr5c00273_si_001.pdf]

SUPPORTING INFORMATION FOR

OCIAD2 promotes cancer progression via metabolic reprogramming in lung adenocarcinoma

Yi-Hui Huang[1,2], Wen-Hsin Chang[3], Chi-Ya Shen[1], Kang-Yi Su[1], Gee-Chen Chang[4], Jin-Shing Chen[5], Wen-Yao Lee[5], Yu-Ju Chen[6], Sung-Liang Yu[1,7,8,9,10,]*

[1]Department of Clinical Laboratory Sciences and Medical Biotechnology, College of Medicine, National Taiwan University, Taipei 10048, Taiwan

[2]Graduate Institute of Medical Genomics and Proteomics, College of Medicine, National Taiwan University, Taipei 100233, Taiwan

[3]Graduate Institute of Medical Science, College of Medicine, Taipei Medical University, Taipei 11031, Taiwan

[4]Department of Internal Medicine, Chung Shan Medical University Hospital, Taichung 40201, Taiwan

[5]Department of Surgery, National Taiwan University Hospital and National Taiwan University College of Medicine, Taipei 10002, Taiwan

[6]Institute of Chemistry, Academia Sinica, Taipei 11529, Taiwan

[7]Department of Laboratory Medicine, National Taiwan University Hospital, Taipei 10002, Taiwan

[8]Institute of Medical Device and Imaging, College of Medicine, National Taiwan University, Taipei 10051, Taiwan

[9]Graduate Institute of Pathology, College of Medicine, National Taiwan University, Taipei 10051, Taiwan

[10]Graduate School of Advanced Technology, National Taiwan University, Taipei, Taiwan, Taipei 10617, Taiwan

***Correspondence:** Sung-Liang Yu, email: slyu@ntu.edu.tw, phone: +886-2-2312-3456 #288697.

CONTENTS

Figure S1. Survival analysis of Taiwan Cancer Moonshot cohort and an independent large-scale proteomic dataset

Figure S2. Overlap of differentially expressed genes identified in public datasets

Figure S3. Quantification and functional assessment of OCIAD2 in lung cancer cells

Figure S4. Exploring the impact of OCIAD2 on mitochondrial pathways

Figure S5. Optimization of the Seahorse Cell Mito Stress Test and normalization of all respiratory parameters

Figure S6. Whole membrane images of the original Western blots

Table S1. Prognostic capability of OCIAD2 on the overall survival of 89 lung adenocarcinoma patients

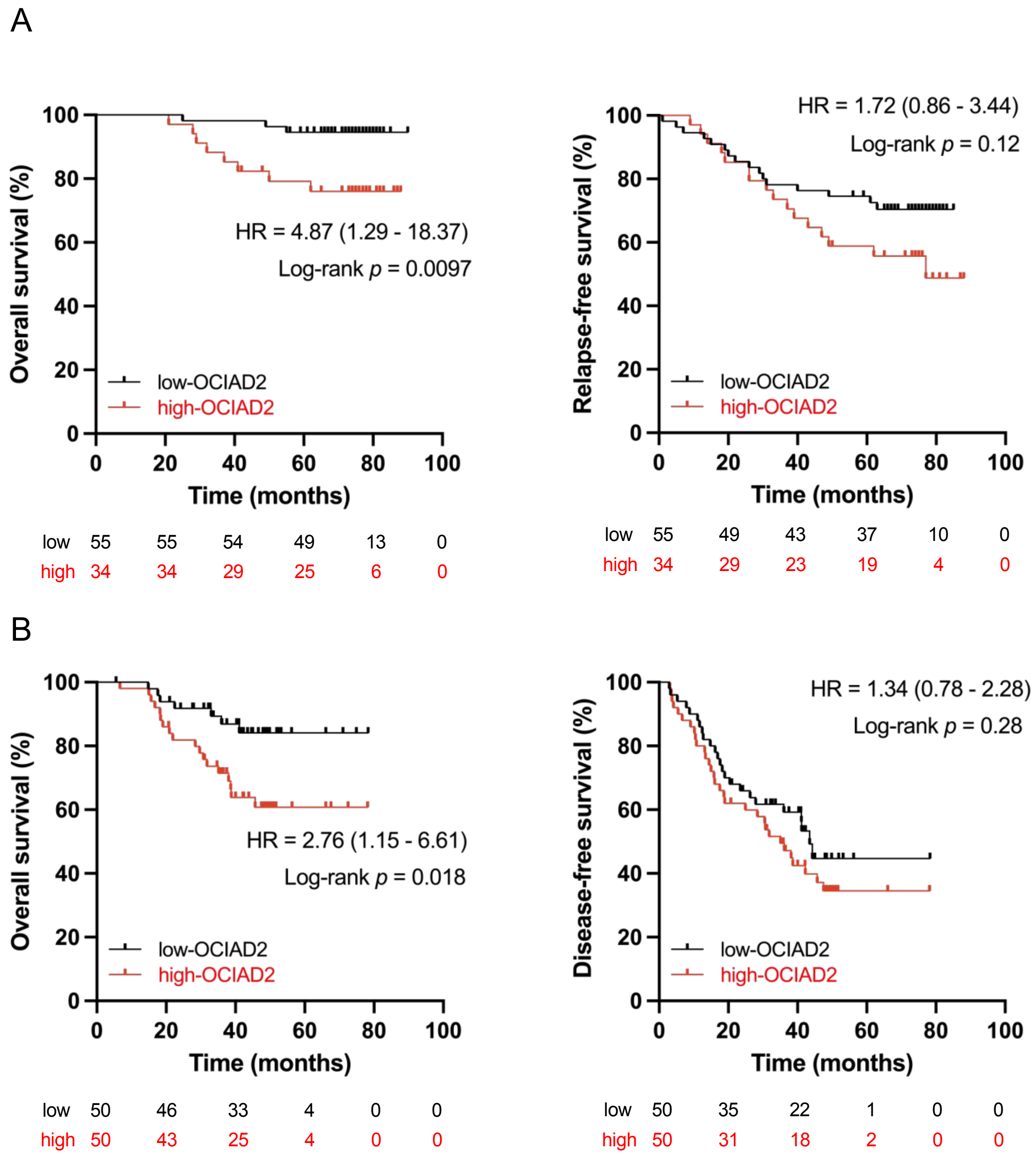

Figure S1. Survival analysis of Taiwan Cancer Moonshot cohort and an independent large-scale proteomic dataset.

(**A**) Kaplan-Meier analysis of overall survival (left) and relapse-free survival (right) for lung adenocarcinoma patients in the TwCM cohort were grouped by high-OCIAD2 and low-OCIAD2 protein expression and compared by the log-rank test. Red line= high-OCIAD2 protein group (patients with Log_2 T/N ratio ≥ 1.055), black line= low-OCIAD2 protein group (patients with

Log_2 T/N ratio < 1.055). (**B**) Survival analysis of overall survival (left) and disease-free survival (right) for lung adenocarcinoma patients in an independent large-scale proteomic dataset, Reference #10 in the main text. Patients were divided into high-OCIAD2 and low-OCIAD2 groups based on their tumor-to-normal (T/N) OCIAD2 expression ratios and compared by the log-rank test.

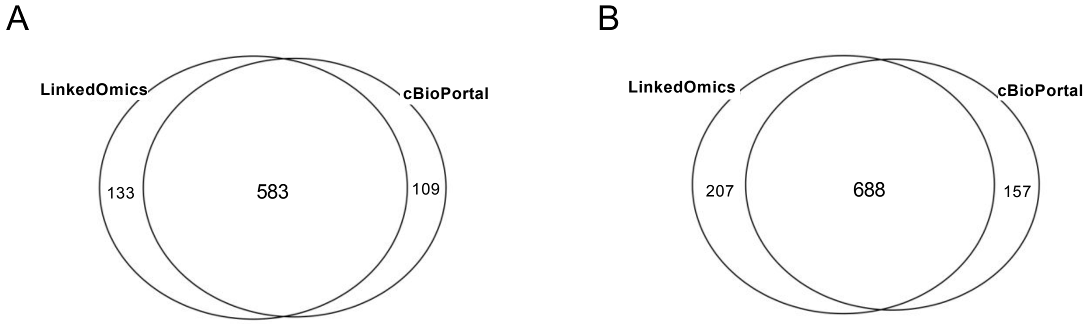

Figure S2. Overlap of differentially expressed genes identified in public datasets.

(**A** and **B**) The Venn diagrams show the overlap of the up-regulated (**A**) and down-regulated (**B**) DEGs between the LinkedOmics and cBioPortal datasets.

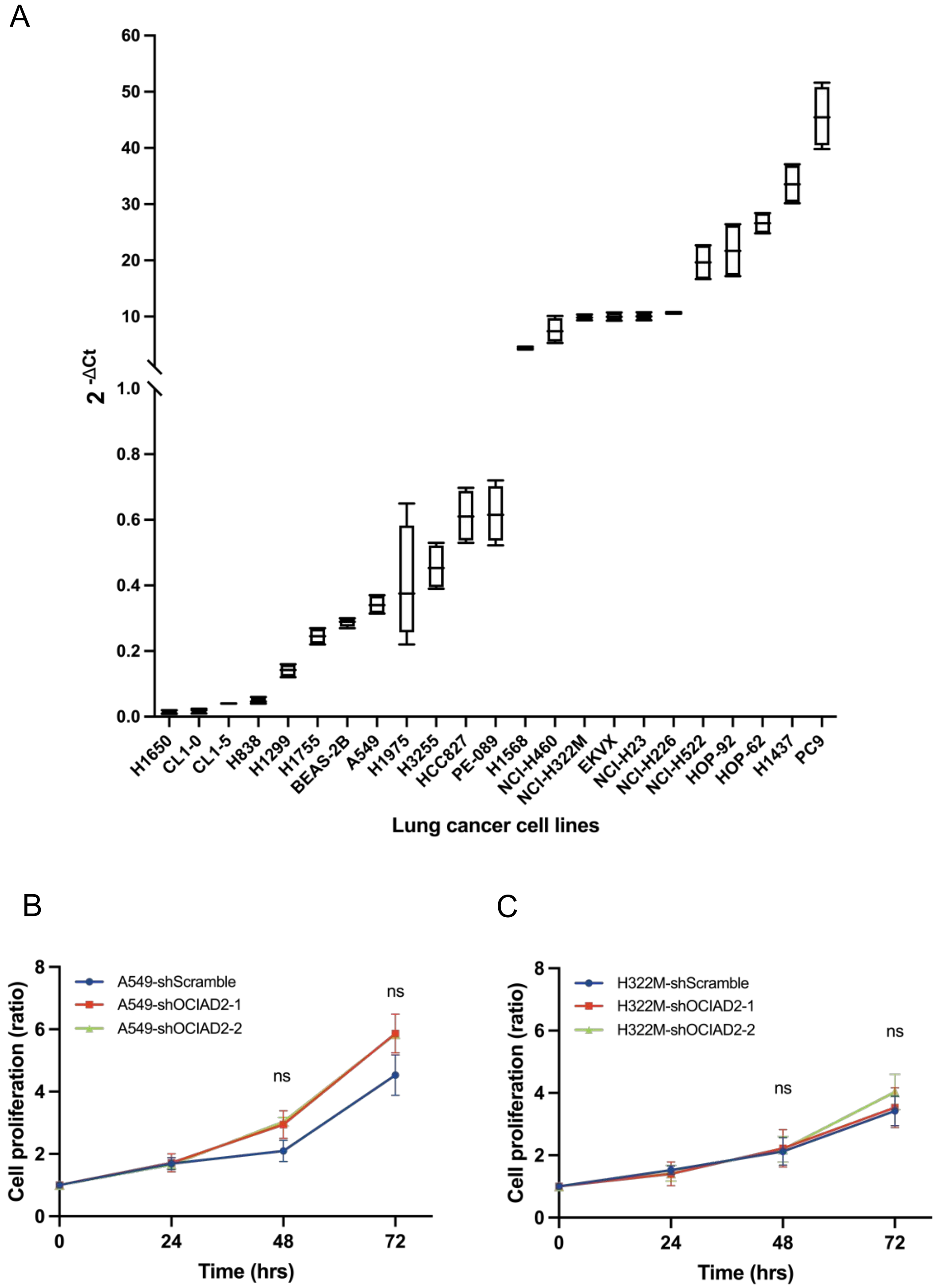

Figure S3. Quantification and functional assessment of OCIAD2 in lung cancer cells.

(**A**) Relative expression of OCIAD2 mRNA in 23 lung cancer cell lines was measured by real-time quantitative RT-PCR and expressed as $2^{-\Delta Ct}$ values. TATA-box binding protein (TBP) serves as the internal control (n = 2). Data are presented as the mean ± SD. (B and C) Cell proliferation in A549 (**B**) and NCI-H322M (**C**) cells was determined by MTT assay (mean ± SD, n = 8).

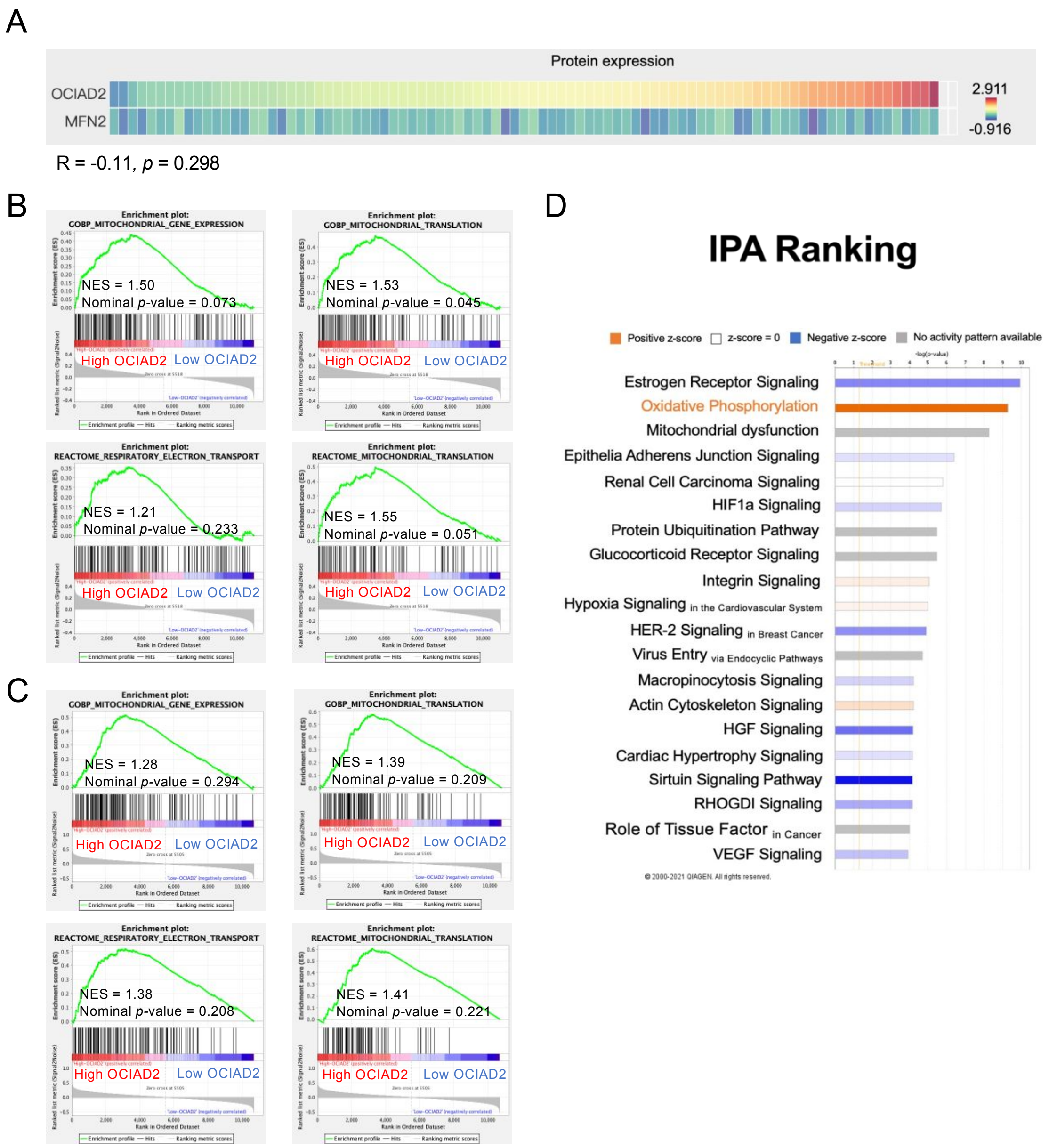

Figure S4. Exploring the impact of OCIAD2 on mitochondrial pathways.

(**A**) OCIAD2 and MFN2 protein expression profiles in the TwCM lung adenocarcinoma cohort, expressed as Log_2 T/N ratios obtained from the Taiwan Cancer Proteogenomic Knowledgebase (https://www.twcms.org/moonshot/portal/index). Each column represents a patient sample. Pearson correlation coefficient (R = -0.11) and p value (p = 0.298) are shown. (**B** and **C**) GSEA

enrichment plots from independent proteomic datasets, Reference #10 (**B**) and Reference #34 (**C**) in the main text. Both panels show mitochondria-related pathways from the GO:BP and CP:Reactome gene sets (corresponding to Fig. 4C-D) and display normalized enrichment scores (NES) and ranking metric scores. (**D**) Ingenuity Pathway Analysis of the co-expressed genes of OCIAD2 in LUAD obtained from the cBioPortal database which identified with a p value<0.05 (y-axis) and | Pearson correlation coefficient (R) | (x-axis) >0.25. The orange column with a positive Z-score connotates predicted pathway activation.

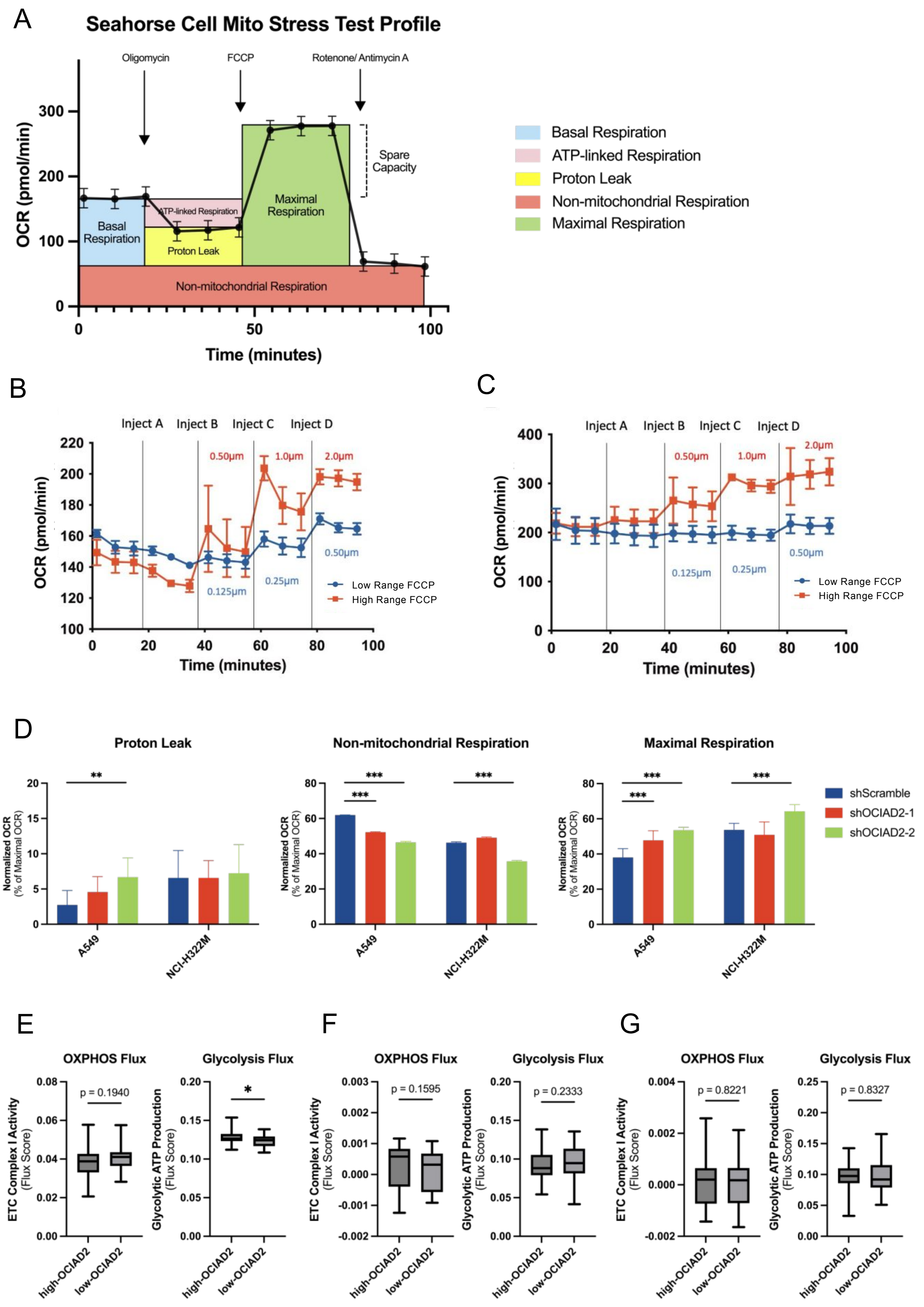
A
Seahorse Cell Mito Stress Test Profile
Oligomycin
FCCP
Rotenone/ Antimycin A
OCR (pmol/min)
Time (minutes)
Basal Respiration
ATP-linked Respiration
Proton Leak
Maximal Respiration
Non-mitochondrial Respiration
Spare Capacity
Basal Respiration
ATP-linked Respiration
Proton Leak
Non-mitochondrial Respiration
Maximal Respiration
B
Inject A
Inject B
Inject C
Inject D
0.50μm
1.0μm
2.0μm
0.125μm
0.25μm
0.50μm
Low Range FCCP
High Range FCCP
C
D
Proton Leak
Non-mitochondrial Respiration
Maximal Respiration
Normalized OCR (% of Maximal OCR)
A549
NCI-H322M
shScramble
shOCIAD2-1
shOCIAD2-2
E
F
G
OXPHOS Flux
Glycolysis Flux
ETC Complex I Activity (Flux Score)
Glycolytic ATP Production (Flux Score)
p = 0.1940
p = 0.1595
p = 0.2333
p = 0.8221
p = 0.8327
high-OCIAD2
low-OCIAD2

Figure S5. Optimization of the Seahorse Cell Mito Stress Test and normalization of all respiratory parameters.

(**A**) The bioenergetics parameters of the Seahorse Cell Mito Stress Test were displayed in an assay profile. (**B** and **C**) Optimization of FCCP dose by injecting Oligomycin (Inject A) and different doses of FCCP (Inject B-D) in A549 shScramble (**B**) and NCI-H322M shScramble cells (**C**). The plate is divided into two groups: a low concentration range (0.125, 0.25, 0.5 μM) and a high concentration range (0.5, 1.0, 2.0 μM). (**D**) Quantification of Proton leak, Non-mitochondrial respiration, and Maximal respiration in A549 and NCI-H322M cells. (**E-G**) Estimated flux scores of OXPHOS (left) and glycolysis (right) functions in lung adenocarcinoma patients from TwCM cohort (**E**) and two large-scale proteomic datasets (**F**: Reference #10; **G**: Reference #34). Each box represents the interquartile range (IQR), and the median value is shown as the central line within each box. The whiskers extend up to 1.5 IQR. *: p value < 0.05.

Figure 3A

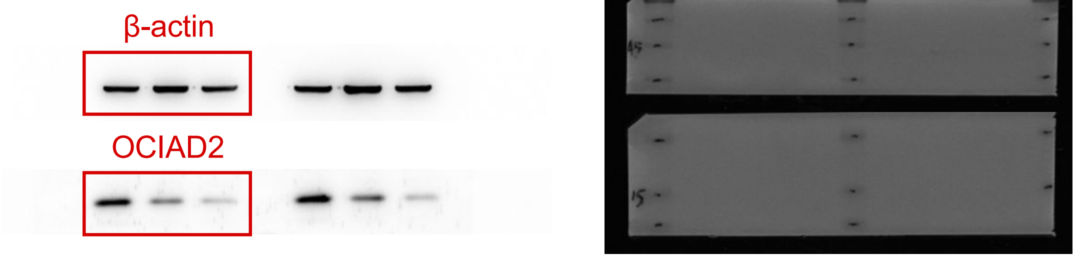

Figure 3C

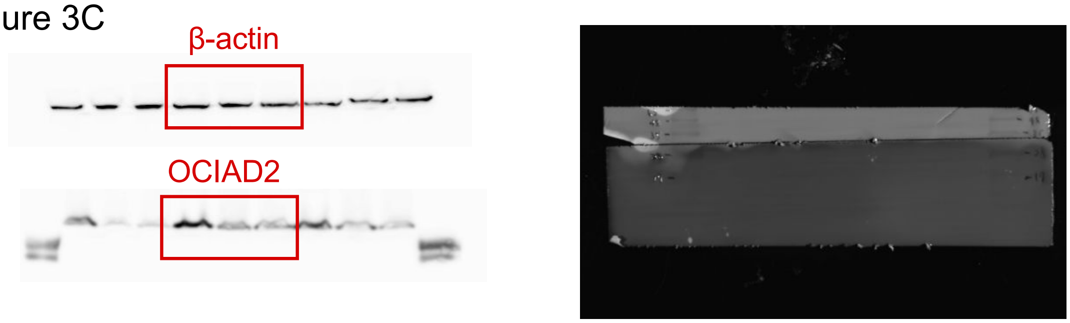

Figure 4A

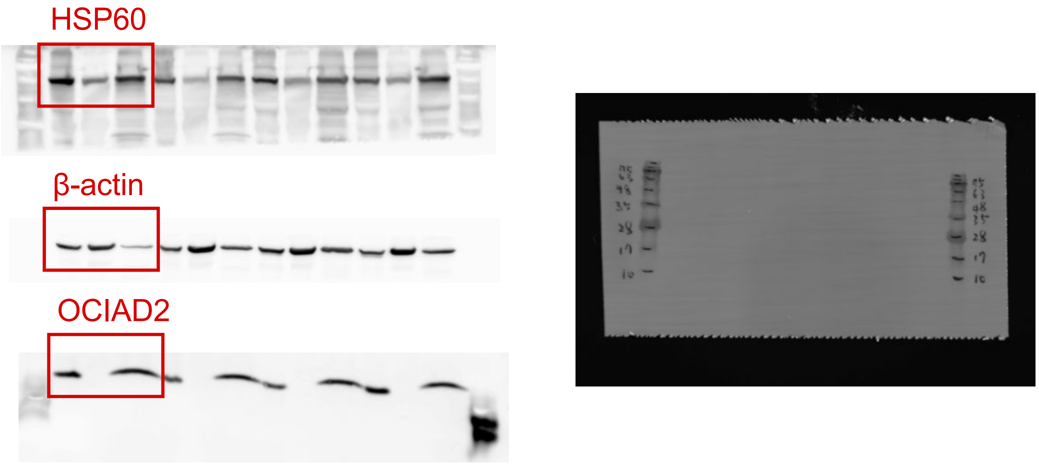

Figure 4B

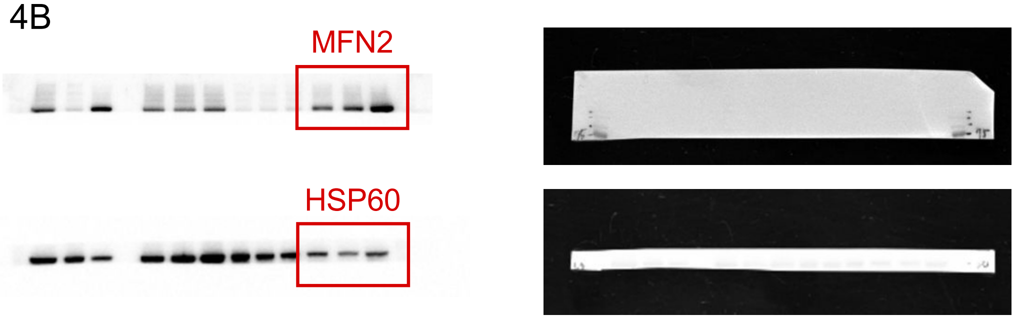

Figure S6. Whole membrane images of the original Western blots.

Images showing the visualized target proteins (left) and the whole membranes under visible light (right) corresponding to Figures 3 and 4. Red squares indicate the lanes presented in the main figures.

Table S1. Prognostic capability of OCIAD2 on the overall survival of 89 lung adenocarcinoma patients

**Log_2 T/N cutoff**	**High-OCIAD2** Patient No.	**Low-OCIAD2** Patient No.	**Log-rank *P* value**
1.4	20	69	**0.037**
1.3	23	66	**0.017**
1.2	29	60	0.094
1.1	33	56	**0.044**
1.055	34	55	**0.0097**
1.05	35	54	**0.013**
1.0	38	51	**0.028**
0.95	40	49	**0.043**
0.9	44	45	0.093
0.8	47	42	0.15
0.7	50	39	0.23
0.6	55	34	0.41
0.5	66	23	0.54
0.4	67	22	0.59
0.38	67	22	0.59
